# Agricultural machinery operation trajectory identification and operation area estimation for cloud-platform deployment

**DOI:** 10.3389/fpls.2026.1874057

**Published:** 2026-07-01

**Authors:** Baozhong Li, Yunhe Feng, Guomin Zhou, Pei Xu, Weipeng Wang

**Affiliations:** 1 Agricultural Information Institute, Chinese Academy of Agricultural Sciences, Beijing, China; 2 Luoyang Smart Agricultural Equipment Institute Co., Ltd., Luoyang, China; 3 National Nanfan Research Institute (Sanya), Chinese Academy of Agricultural Sciences, Sanya, China; 4 Nanjing Institute of Agricultural Mechanization, Ministry of Agriculture and Rural Affairs, Nanjing, China

**Keywords:** agricultural machinery, buffer algorithm, operation area, operation trajectory, semantic segmentation

## Abstract

Precise identification of agricultural machinery operation trajectories and efficient estimation of operation area are essential for cloud-platform-based machinery supervision and service settlement. However, raw GNSS trajectories collected from practical operation platforms are often affected by positioning drift, missing points, road-transfer trajectories, headland turns, and repeated or pseudo-missed operations, making it difficult for either purely rule-based trajectory screening or direct buffer-based area recovery to simultaneously achieve reliable recognition accuracy and engineering efficiency. To address this problem, this study proposes a staged trajectory identification and area estimation method for cloud-platform deployment. The method first standardizes raw trajectory sequences through attribute integrity checking, motion rationality filtering, speed cleaning, and temporal interpolation. Candidate operation trajectories are then organized using spatiotemporal neighborhood constraints to remove evidently non-operational long-distance transfer trajectories before image construction. A multi-channel trajectory image representation, in which speed, acceleration, and heading variation are encoded as feature channels, is further used as the input of an improved CBGAM U-Net semantic segmentation model for pixel-level refinement of field-operation trajectories. Finally, a cubic-spline-smoothed vector-buffer algorithm with width compensation and inward boundary correction is used to reconstruct operation coverage areas. Experimental results on the IAEMP platform dataset showed that the proposed method achieved an average trajectory recognition accuracy of 96.32%. In parcel-level area validation, the absolute area estimation errors of the tested field parcels were controlled within 3.00%, and the cloud-platform deployment test showed stable processing efficiency for practical operation records. In independent Real-Time Kinematic (RTK)-based field validation, the area accuracies reached 99.64% for wheat harvesting and 99.91% for rotary tillage. These results demonstrate that the proposed staged framework can improve trajectory identification reliability and area estimation consistency while maintaining practical deployability for agricultural machinery management platforms.

## 1 Introduction

With the continuous advancement of agricultural mechanization and informatization, cloud-platform-based agricultural machinery operation management has gradually become an important support for modern agricultural production organization and service management (Stafford, 2000). Relying on onboard terminals, wireless communication networks, and the Global Navigation Satellite System (GNSS), information such as trajectories, speed, heading, and operation status can be continuously acquired during machinery operation and then centrally stored, uniformly analyzed, and visually presented on the cloud platform (Zhang et al., 2002). Agricultural machinery operation trajectory data not only reflect the spatiotemporal behavioral characteristics of machinery operation, but also have important application value in operation supervision, operation quality assessment, operation scheduling, and machinery service management (Köksal and Tekinerdogan, 2018). In large-scale operation management scenarios in particular, the extraction of reliable operational information from massive trajectory datasets has become a key prerequisite for intelligent and refined services on agricultural machinery management platforms (Zhang et al., 2022).

Among various indicators used in agricultural machinery operation management, operation area is one of the core quantitative metrics for characterizing operation scale, operation effectiveness, and task completion (Luo et al., 2022). Its accurate estimation directly affects the validity of operation statistics, supervision, service settlement, and platform-level decision-making. Compared with manual reporting and experience-based estimation, automatic area estimation based on GNSS trajectory data offers several advantages, including high timeliness, wide coverage, and low deployment cost, and has therefore been widely adopted in agricultural machinery operation management platforms (Wu et al., 2020). However, in practical data acquisition, raw machinery trajectories are often affected by GNSS positioning drift, terminal anomalies, communication interruptions, and frequent switching of operation states, resulting in widespread problems such as noisy points, sampling discontinuities, mixed road-transfer trajectories, and interference from non-operational behaviors (Radočaj et al., 2023). Under such conditions, direct area calculation from raw trajectories can easily lead to distorted operation boundaries and inaccurate area estimates (Guo et al., 2018; Zheng, 2015). Therefore, the accuracy of operation area estimation depends to a large extent on the reliable identification of valid operation trajectories.

Existing methods for agricultural machinery operation area calculation can be broadly classified into three categories (Lu et al., 2015). The first category performs geometric calculation or area recovery directly from raw trajectories (Poteko et al., 2021). Although this approach is relatively simple to implement, it is highly sensitive to positioning drift, trajectory discontinuities, and mixed road-transfer trajectories, causing errors to accumulate rapidly under complex conditions. The second category relies on rules or thresholds to screen and classify trajectories, for example by distinguishing operation trajectories from transfer trajectories using trajectory density, speed, heading variation, and local geometric features. Such methods are convenient to implement and involve relatively low engineering deployment cost, but their performance is usually highly dependent on empirically selected parameters and remains limited in robustness under complex scenarios such as field boundaries, headland turns, road turns, and field-road intersections. The third category reconstructs operation areas through vector buffering or geometric coverage (Ren et al., 2017), which naturally converts line-based trajectories into area-based operation regions and is therefore suitable for engineering implementation and batch processing. However, in the absence of reliable constraints on valid operation trajectories and necessary boundary correction, these methods are prone to systematic overestimation caused by long-distance false connections, positioning bias, and buffer expansion effects. Accordingly, the key issue is not merely how to calculate operation area, but rather how to accurately identify valid operation trajectories from complex operational trajectories and, on that basis, construct a geometrically sound area recovery method.

In recent years, the rapid development of deep learning in image understanding and pattern recognition has provided a new perspective for operation trajectory identification under complex trajectory scenarios (Zhai et al., 2024b). Compared with methods that rely solely on point-level spatiotemporal rules, semantic segmentation can characterize target shapes and boundaries at the pixel level while fully exploiting both local features and global contextual information, thereby exhibiting stronger representation capability in complex boundary regions and mixed-trajectory discrimination. For agricultural machinery trajectories (Han et al., 2025; Zhang et al., 2024; Zhai et al., 2024a), motion attributes such as speed, acceleration, and heading variation can be further mapped to pixel values in trajectory images, transforming the trajectory identification task from simple point-sequence discrimination into an image segmentation problem that integrates motion information with spatial structural information (Shelhamer et al., 2017; Chen et al., 2018). However, purely rule-based methods are limited in their discriminative capability under complex scenarios, whereas purely deep-learning-based methods may suffer from high computational cost and strong dependence on training samples. Therefore, for agricultural machinery operation management platforms, there remains a need for a staged methodological framework that jointly considers engineering efficiency, robustness in complex scenarios, and feasibility for online deployment.

Although the above methods have improved the automatic extraction of agricultural machinery operation information, a persistent trade-off remains between recognition accuracy and computational efficiency in cloud-platform scenarios. Geometric and rule-based methods are computationally efficient and easy to deploy, but their performance is sensitive to manually selected thresholds and is easily degraded when field-operation trajectories are mixed with road-transfer trajectories, headland turns, static drifting, and sparse sampling segments. Deep-learning-based methods provide stronger representation capability for complex spatial patterns and field boundaries, but applying them directly to raw large-span trajectory records increases computational cost and may introduce irrelevant road-transfer information into the image representation. Buffer-based area recovery is also efficient and intuitive, but it is highly dependent on the correctness of the extracted trajectory centerlines. Therefore, the key challenge is not simply to replace traditional methods with a single deep-learning model, but to construct a staged framework in which each method is used only for the problem scale and data representation to which it is best suited.

To address this challenge, this study proposes a cloud-platform-oriented framework that links trajectory identification with operation area estimation through a staged constraint-transfer strategy. Rather than simply combining preprocessing, clustering, semantic segmentation, and buffering, the proposed method assigns each stage a distinct role in progressively narrowing the uncertainty of raw GNSS trajectories: preprocessing regularizes noisy and discontinuous point sequences, spatiotemporal grouping removes evident long-distance transfer trajectories and reduces the spatial scope of subsequent analysis, semantic segmentation focuses on ambiguous mixed regions that cannot be reliably separated by rules alone, and vector-buffer reconstruction estimates the operation area only from the refined valid trajectories. Based on this framework, the main contributions of this study are summarized as follows: (1) a cloud-platform-oriented multi-stage trajectory identification framework is established to progressively refine mixed agricultural machinery trajectories from point-sequence constraints to pixel-level semantic discrimination; (2) multi-channel trajectory image representation and an improved CBGAM U-Net are developed to enhance the discrimination of field-operation, road-transfer, drifting, and field-road intersection trajectories; and (3) a cubic-spline-smoothed vector-buffer area estimation method with width compensation and inward boundary correction is proposed to reduce area bias caused by sparse sampling, positioning deviation, and local trajectory irregularity. The proposed framework is further validated using historical cloud-platform records and independent RTK-based field measurements, demonstrating its applicability to both engineering deployment and geometric area estimation.

## 2 Materials and methods

### 2.1 Agricultural machinery cloud platform for operation management

#### 2.1.1 Cloud platform architecture

**Figure 1:**
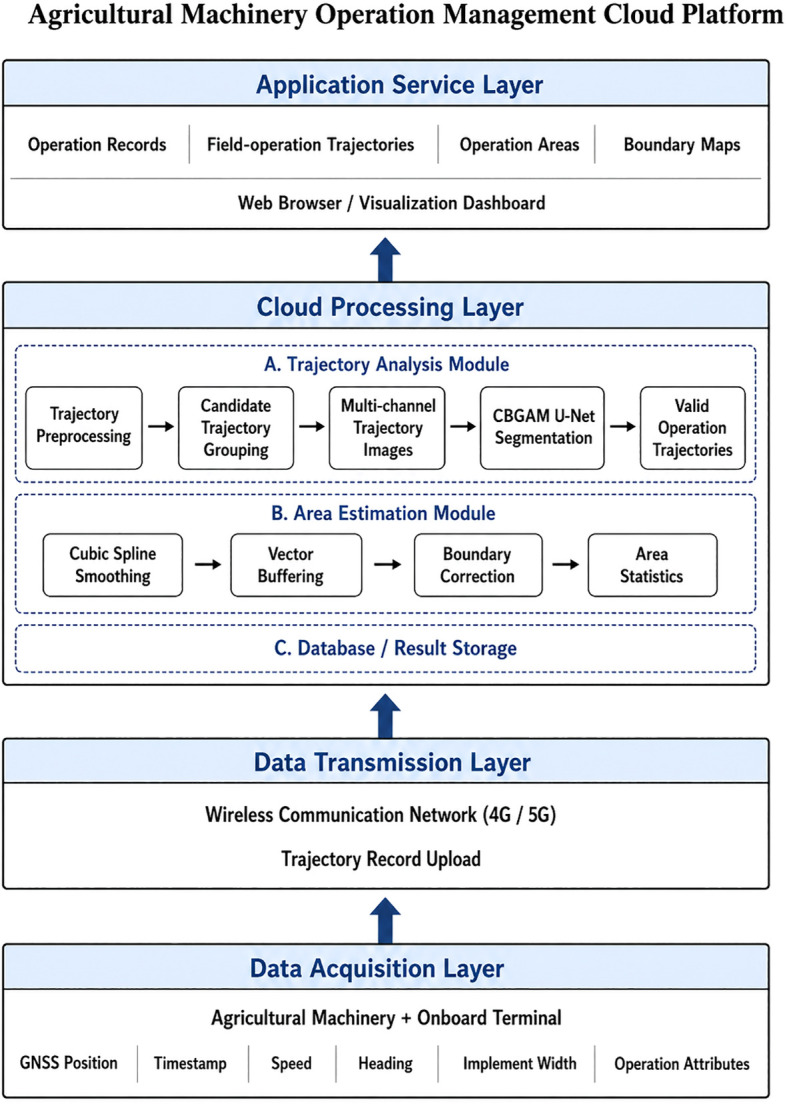
Architecture of agricultural machinery operation management platform.

To support the centralized management, online analysis, and visual presentation of large-scale agricultural machinery operation trajectory data, a cloud platform for agricultural machinery operation management was developed, and the proposed trajectory identification and operation area estimation methods were implemented within this platform, as shown in **Figure 1**. The platform adopts a Browser/Server (B/S) architecture and consists of four functional layers: the data acquisition layer, data transmission layer, cloud processing layer, and application service layer. The data acquisition layer is composed of onboard terminals installed on agricultural machinery and is responsible for collecting GNSS positions, timestamps, speed, heading, implement width, and other operation-related attributes. The data transmission layer uploads the collected trajectory records to the cloud server through wireless communication networks and provides the data stream required for subsequent processing. The cloud processing layer is the core analytical layer of the platform, where trajectory preprocessing, candidate trajectory grouping, semantic-segmentation-based refinement, vector-buffer-based area estimation, and result storage are performed. The application service layer provides users with visualized outputs, including operation records, identified field-operation trajectories, estimated operation areas, and field boundary contours.

#### 2.1.2 Trajectory data ingestion and management

**Figure 2:**
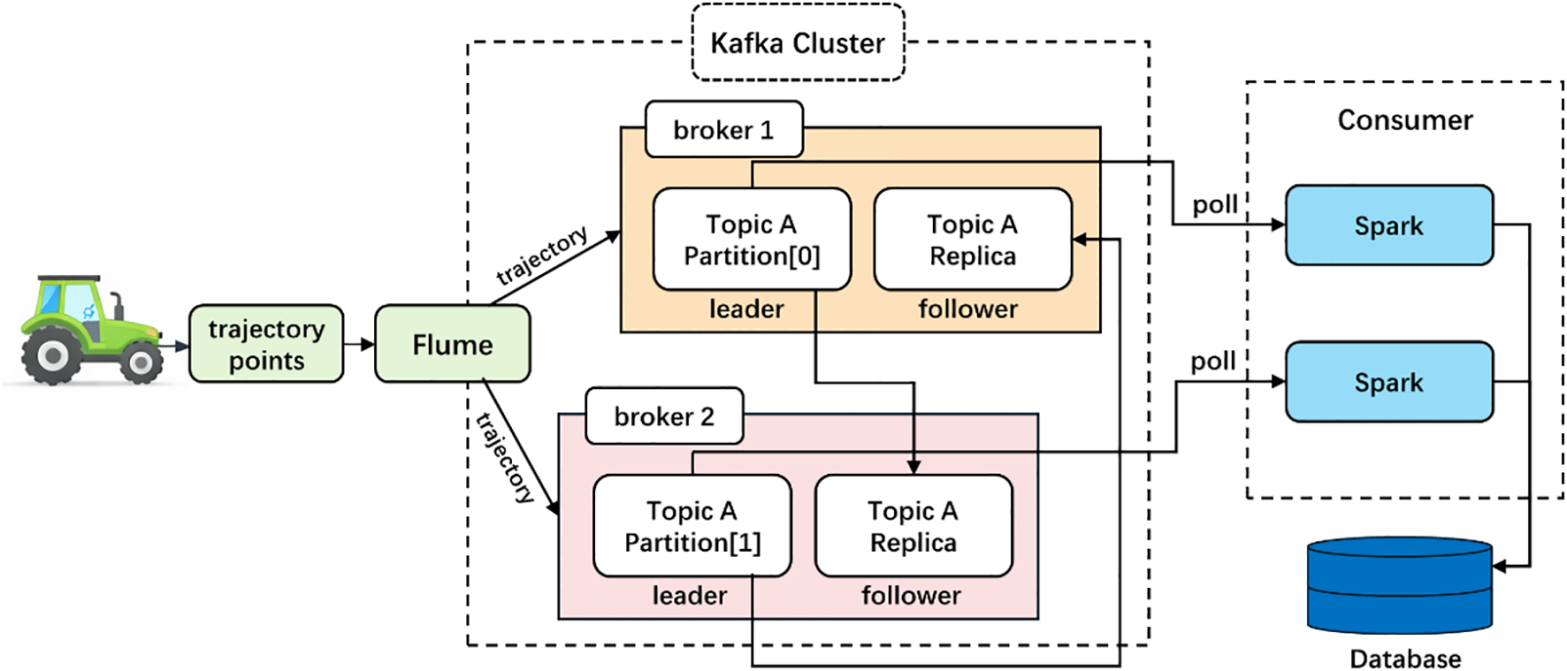
Trajectory data management.

For trajectory data organization and management, the platform employs a unified data ingestion and management mechanism for continuously uploaded trajectory data from onboard terminals, thereby ensuring structural consistency across different agricultural machines, implements, and operational tasks. To meet the requirements of high-concurrency data acquisition and real-time processing, Apache Kafka was adopted as a distributed message middleware to construct the data streaming pipeline. As a high-throughput publish–subscribe messaging system, Kafka classifies and stores massive trajectory data generated by GNSS positioning modules through a topic and partition-based mechanism, thereby decoupling data producers from consumers. In the system architecture, as shown in **Figure 2**, Flume acts as the producer and writes agricultural machinery trajectory data into designated topics, while Spark acts as the consumer and asynchronously retrieves data through a subscription mechanism. This design not only ensures the timeliness of data acquisition but also reduces the risk of data loss through persistent message storage.

#### 2.1.3 Overall technical framework for operation trajectory identification and area estimation

**Figure 3:**
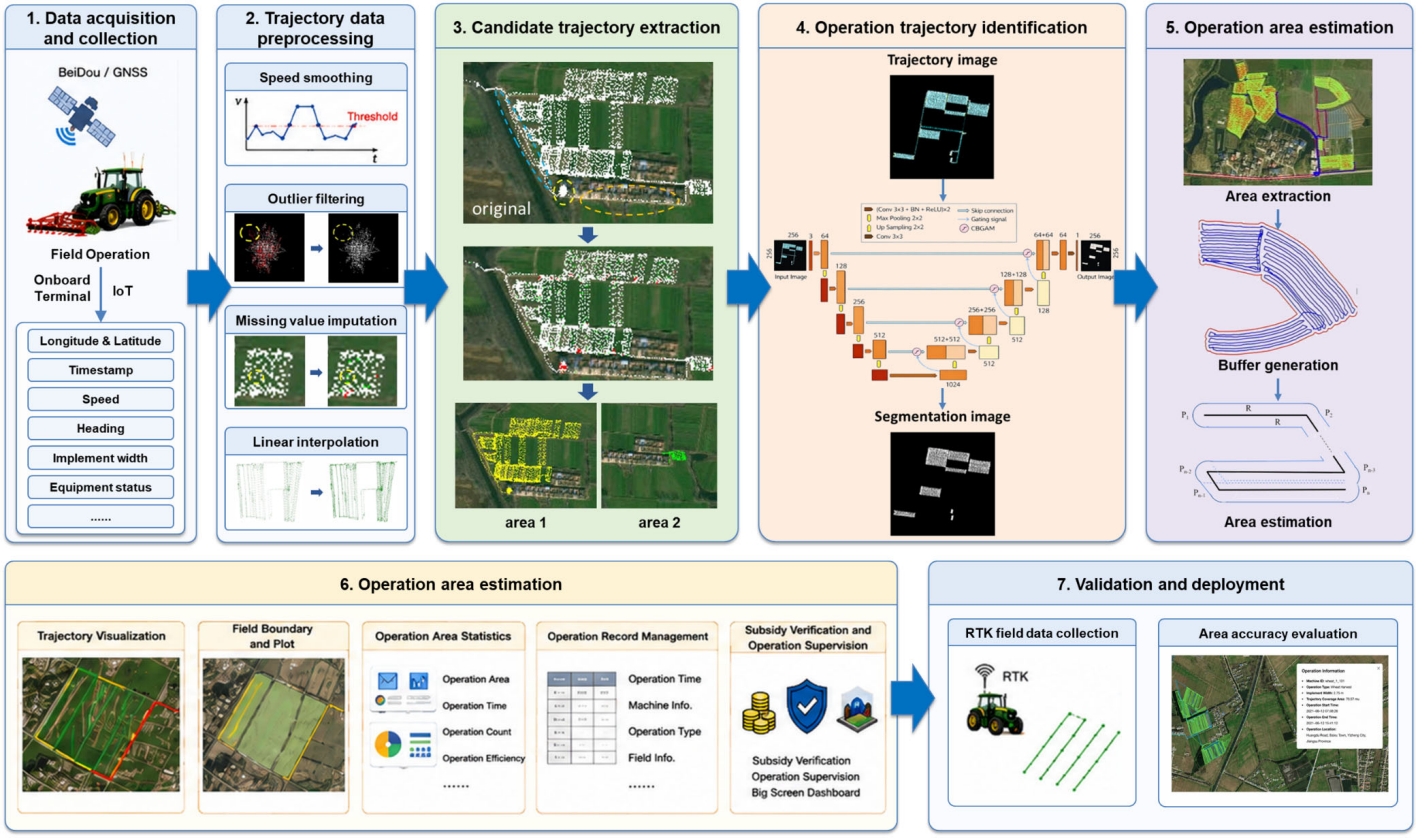
Overall technical framework for operation trajectory identification and area estimation.

The technical framework for agricultural machinery operation trajectory identification and operation area estimation was established, as illustrated in **Figure 3**. The framework integrates trajectory acquisition, data preprocessing, candidate trajectory extraction, operation trajectory identification, operation area estimation, platform-level application, and field validation into a unified technical pipeline. Through this workflow, raw GNSS trajectory records collected from agricultural machinery are progressively transformed into parcel-level operation boundaries and operation area statistics that can be directly deployed within the cloud platform.

The framework consists of five sequential stages. First, raw trajectory records uploaded from onboard terminals are standardized through missing-value handling, attribute filtering, speed cleaning, and temporal interpolation to improve data completeness and temporal continuity. Second, spatiotemporal neighborhood constraints are employed to group candidate trajectory segments and remove evidently non-operational long-distance transfer trajectories, thereby reducing trajectory noise and the spatial extent of subsequent processing. Third, the retained candidate trajectories are converted into multi-channel trajectory images by encoding spatial coordinates, travelling speed, local acceleration, and heading variation. The CBGAM U-Net model is then used to perform semantic segmentation and identify valid field-operation trajectories under complex scenarios, including field-road intersections, road turns, drifting trajectories, and mixed operation regions. Fourth, the refined operation trajectory centerlines are reconstructed into operation coverage polygons through cubic spline smoothing, width-compensated vector buffering, polygon union, and inward boundary correction, from which operation areas are calculated. Finally, the identified operation trajectories, operation boundaries, and area statistics are integrated into the cloud platform for visualization and management, while independent RTK-based field measurements are used to evaluate the accuracy of the estimated operation areas.2.2 Dataset and task definition.

The dataset used for model development and platform-level testing was the Intelligent Agricultural Equipment Management Platform (IAEMP) dataset collected from the Smart Agricultural Machinery Management Platform, as shown in **Table 1**. The IAEMP dataset is not a public benchmark dataset, but a historical operation dataset generated by onboard terminals deployed on agricultural machinery during practical production operations. The dataset contains 3,732 operation trajectories with 843,200 trajectory points. The data were collected from 18 provinces in China, including Henan and Heilongjiang, and cover 24 types of agricultural machinery, such as rice transplanters, wheat harvesters, corn harvesters, and tillage machines. Approximately 94% of the trajectory points were recorded at a sampling interval of 2s. The IAEMP dataset was used for trajectory-image construction, model training/testing, and platform-level performance evaluation.

**Table 1:** IAEMP dataset.

Parameters	IAEMP dataset
Sampling platform	Smart Agricultural Machinery Management Platform
Sampling period	2023.5.23-2024.10.20
Number of trajectory	3,732
Covered provinces	18 (Henan, Heilongjiang, etc.)
Agricultural machinery	24 (rice transplanter, wheat harvester, corn harvester, tillager, etc.)
Number of trajectory point	843,200
Sampling frequency	Record 94% of trajectory points every 2 seconds

To formulate agricultural machinery operation trajectory identification as a supervised learning task, trajectory labels were constructed through manual annotation. Each JSON trajectory record was first projected onto a satellite map and checked together with its temporal sequence, speed, heading angle, implement width, and operation log information when available. The actual operation region was manually delineated by trained annotators according to the visible parcel boundary, reciprocating operation pattern, trajectory density, and operation continuity. Trajectory points located within the delineated operation region and corresponding to effective field operation were labeled as operation points, whereas points corresponding to road transfer, static drift, parking, obvious GNSS outliers, road turns, and non-working headland movements were labeled as non-operation points.

For ambiguous cases, the annotation followed a conservative rule. Headland turns were labeled as operation points only when they occurred inside the field boundary and were part of a continuous operation pass or necessary turning process for the same operation task. Headland movements, road turns, or transition trajectories were labeled as non-operation points when they occurred outside the field boundary, connected different parcels, or did not contribute to actual implement coverage. Samples with serious positioning disorder, incomplete operation records, or annotation disagreement after review were excluded from the training set.

**Figure 4:**
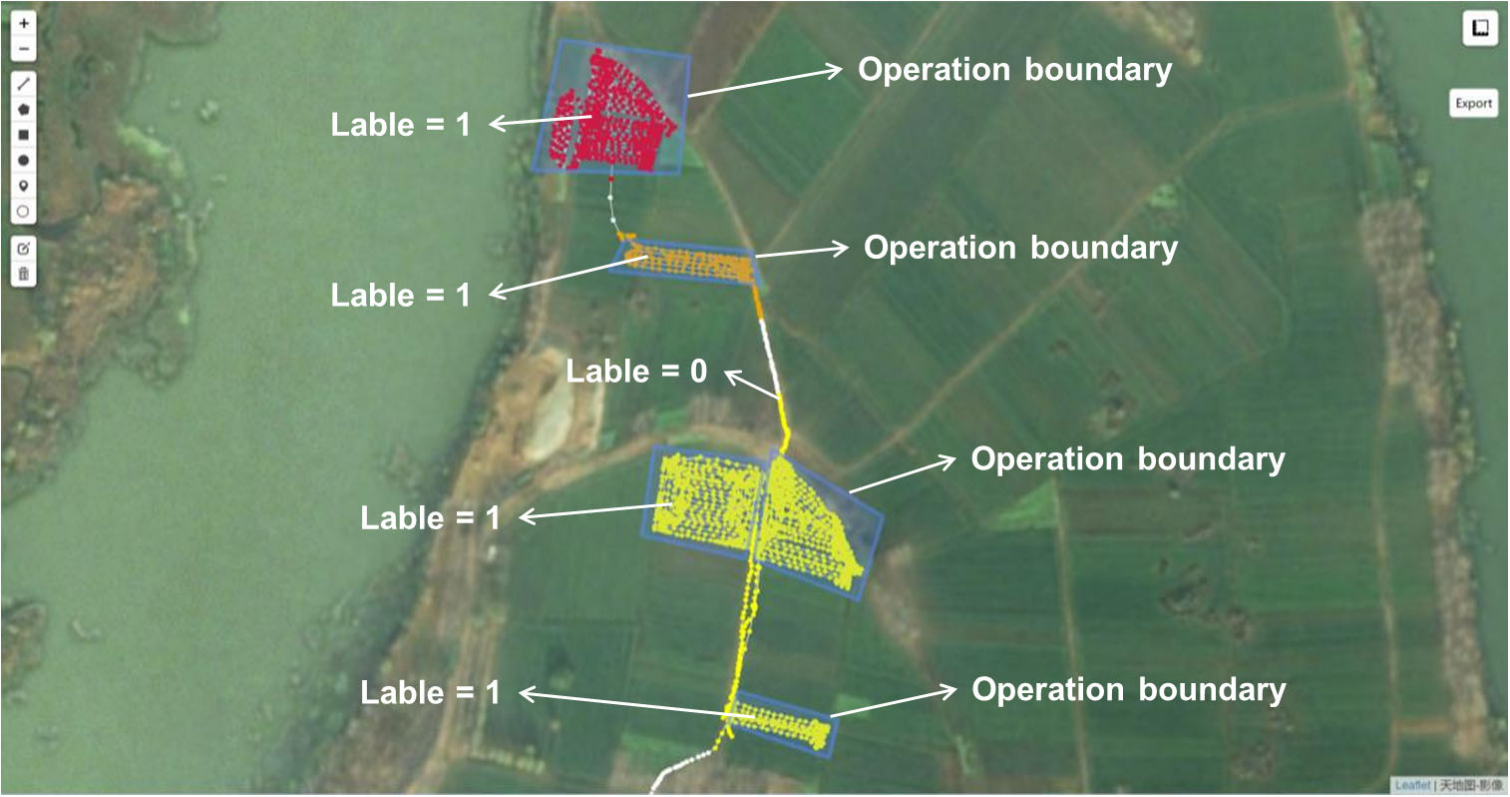
Trajectory labelling.

In the adopted labeling scheme, operation points were assigned a value of “1” and non-operation points a value of “0”, which together constitute a binary classification system, as shown in **Figure 4**.

Accordingly, the class label of an individual trajectory point can be defined as Equation 1:

(1) $$y_{i}\in \left\{0,1\right\}$$

Where, *y_i_* = 1 indicates that the trajectory point belongs to a valid field operation trajectory, and *y_i_* = 0 indicates that it belongs to a road-transfer, drifting, turning, or other non-operational trajectory. Then, a single operation task can be represented Equation 2 as a labeled trajectory sequence *τ*:

(2) $$\tau ={\left\{\left(p_{i},y_{i}\right)\right\}}_{i=1}^{N}$$

Where, *p_i_* denotes the original attribute vector of the *i*_th_ trajectory point. Therefore, as illustrated in **Figure 4**, the task addressed in this study is defined as follows: for a given raw trajectory sequence *τ*, a trajectory identification function is first applied to extract the valid operation trajectory set *τ*_op_ from the mixed spatiotemporal sequence containing both operational and non-operational trajectories; subsequently, an area estimation function is used to map the valid operation trajectories to the final operation area *S*.

### 2.3 Multi-stage operation trajectory identification

**Figure 5:**
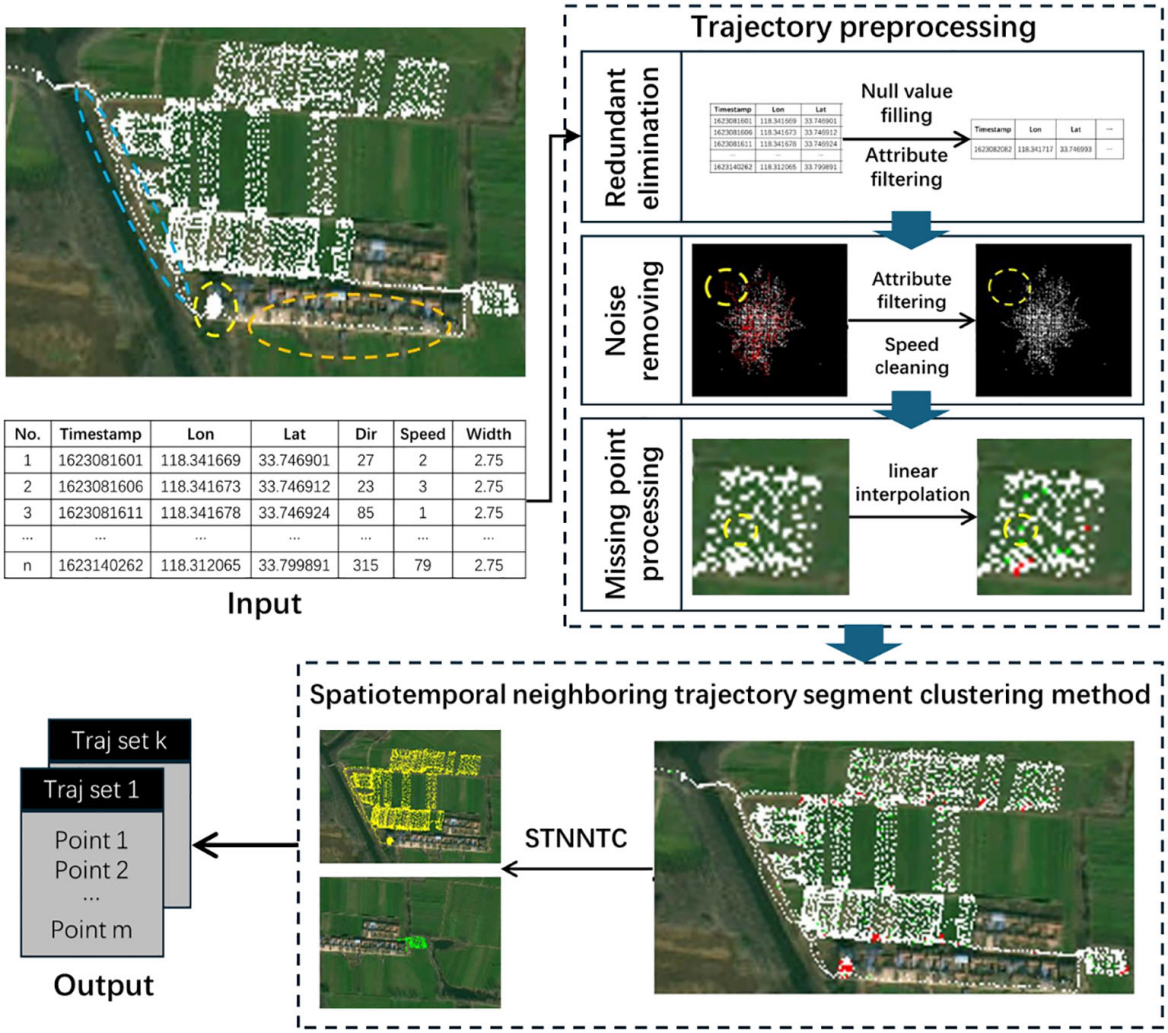
Multi-stage operation trajectory identification method.

To improve the accuracy of agricultural machinery operation trajectory identification under complex scenarios, a multi-stage operation trajectory identification method was developed, as shown in **Figure 5**. Using daily machinery operation trajectories as input, the method integrates the spatiotemporal distribution characteristics of trajectory points, local kinematic features, and the global spatial representation capability of trajectory images. Candidate operation trajectories are first grouped and screened through trajectory preprocessing and spatiotemporal neighborhood constraints, after which complex mixed trajectories are further refined using a semantic segmentation model to extract valid operation trajectories. Long-distance road-transfer trajectories usually span a large spatial range. If they are directly mapped together with field operation trajectories into trajectory images, local pixel-level overlap between field and road trajectories can occur, thereby weakening the ability of subsequent semantic segmentation to characterize boundary and shape features. Candidate trajectories were therefore first organized, screened, and grouped at the trajectory-segment level before trajectory image construction and semantic segmentation were performed, so as to balance computational efficiency and identification robustness under complex scenarios.

Let the raw trajectory sequence of a single operation task be denoted as Equation 3:

(3) $$\tau ={\left\{p_{i}\right\}}_{i=1}^{N}\text{, }p_{i}=\left(\lambda_{i},\phi_{i},t_{i},v_{i},\theta_{i},w_{i}\right)$$

Where λ*_i_* and *ϕ_i_* denote the longitude and latitude of the *i*_th_ trajectory point, *t_i_* denotes the timestamp, *v_i_* denotes the travelling speed, *θ_i_* denotes the heading angle, *w_i_* denotes the implement working width. Since distance calculation, buffer construction, and area estimation are all performed in a planar space, the longitude and latitude are first transformed into planar coordinates through a coordinate projection. Thus, the planar trajectory point ${\tilde{p}}_{i}$ can be expressed as Equation 4:

(4) $${\tilde{p}}_{i}=\left(x_{i},y_{i},t_{i},v_{i},\theta_{i},w_{i}\right)$$

and the planar trajectory sequence $\tilde{\tau }$ can be expressed as Equation 5:

(5) $$\tilde{\tau }=\left\{{\tilde{p}}_{i}\right\}$$

#### 2.3.1 Multi-stage trajectory preprocessing

Affected by terminal anomalies, satellite positioning errors, communication interruptions, and complex operating environments, raw trajectories often contain missing values, duplicate points, static drifting points, abnormal positioning points, and overspeed points. Directly using such raw trajectories for classification and area estimation can easily lead to trajectory breaks, false connections, and boundary distortion. Therefore, a preprocessing operator is applied to the raw trajectory sequence to obtain a regularized trajectory sequence $\tau^{\left(0\right)}$, expressed as follows Equation 6:

(6) $$\tau^{\left(0\right)}={\text{Φ}}_{pre}\left(\tilde{\tau }\right)$$

**Figure 6:**
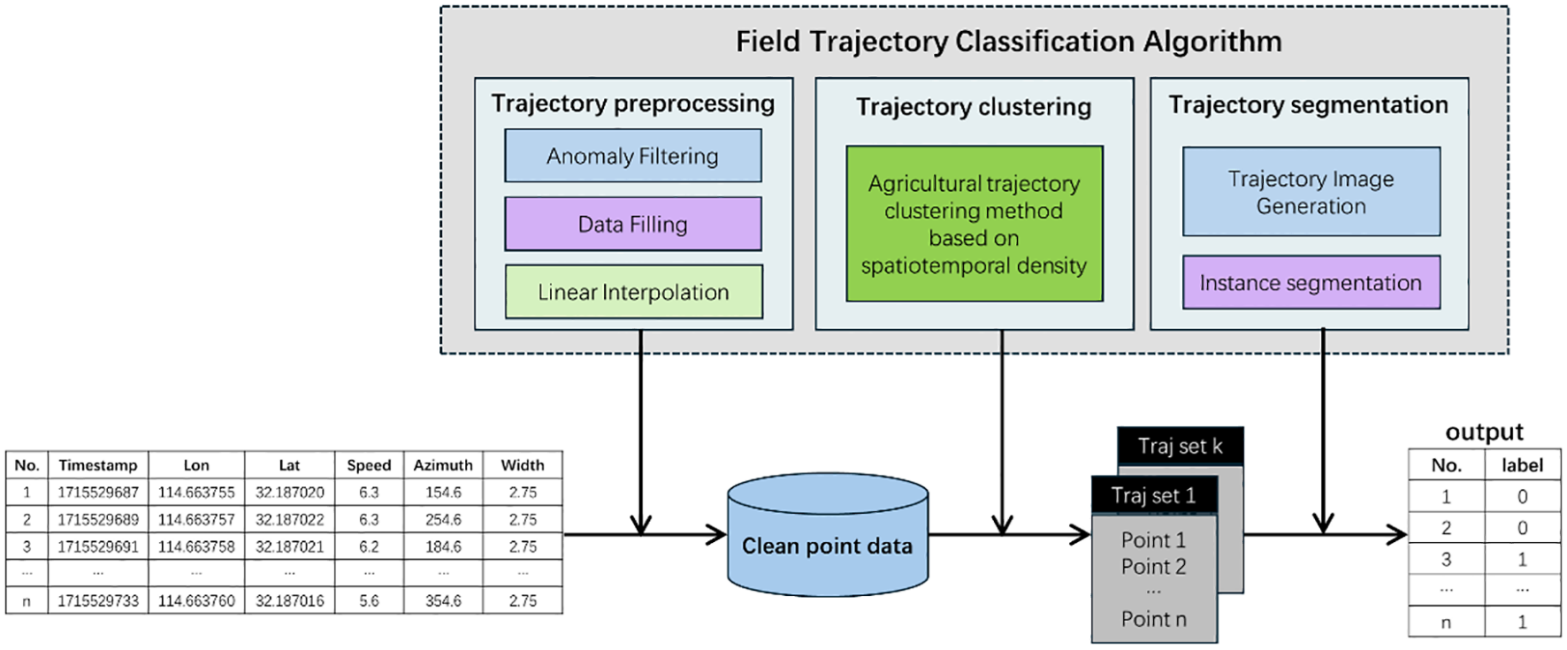
Trajectory preprocessing.

The preprocessing procedure can be functionally summarized into three aspects, as shown in **Figure 6**: attribute integrity constraints, motion rationality constraints, and temporal continuity restoration. In implementation, these correspond to four specific operations, namely missing-value handling, attribute filtering, speed cleaning, and linear interpolation. For trajectory points with missing critical attributes, the type of missingness is first identified, and completion, placeholder assignment, or subsequent removal is applied according to the attribute category to ensure the computational validity of the sequence structure. For points with duplicated timestamps, duplicated positions, or evidently abnormal attributes, redundant and invalid points are removed through attribute filtering. For trajectory points with abnormal speeds, cleaning is performed according to the maximum reasonable operating speed threshold of the corresponding machinery type, thereby reducing the interference of overspeed points with subsequent trajectory organization. For irregular temporal intervals caused by short-term communication interruptions or sampling gaps, intermediate trajectory points are restored by linear interpolation under the assumption of temporal continuity. After preprocessing, the raw trajectories are transformed into a standardized trajectory sequence with temporal continuity, controlled noise, and complete structure, providing input for the subsequent grouping and screening of candidate operation trajectories.

Under attribute integrity constraints, trajectory points with missing key attributes, such as timestamps, positions, and speeds, are first identified, and removal, placeholder assignment, or interpolation is applied according to the attribute type to ensure the computational tractability of subsequent sequence processing. Redundant points with duplicated positions and duplicated timestamps are directly removed. Abnormal positioning points that clearly fall outside the study area are eliminated by incorporating regional boundary constraints.

Under motion rationality constraints, a maximum reasonable operating speed threshold ${\text{v}}_{\max}^{\left(\text{m}\right)}$ is specified according to the machinery type, and trajectory points satisfying $0\leq {\text{v}}_{\text{i}}\leq {\text{v}}_{\max}^{\left(\text{m}\right)}$ are regarded as valid candidate points. Trajectory points exceeding this range are identified as abnormal and removed. In addition, for static drifting trajectories, auxiliary identification is performed by jointly considering the displacement between consecutive points and the local average speed, thereby reducing the interference of non-operational stationary states with subsequent trajectory image construction.

**Figure 7:**
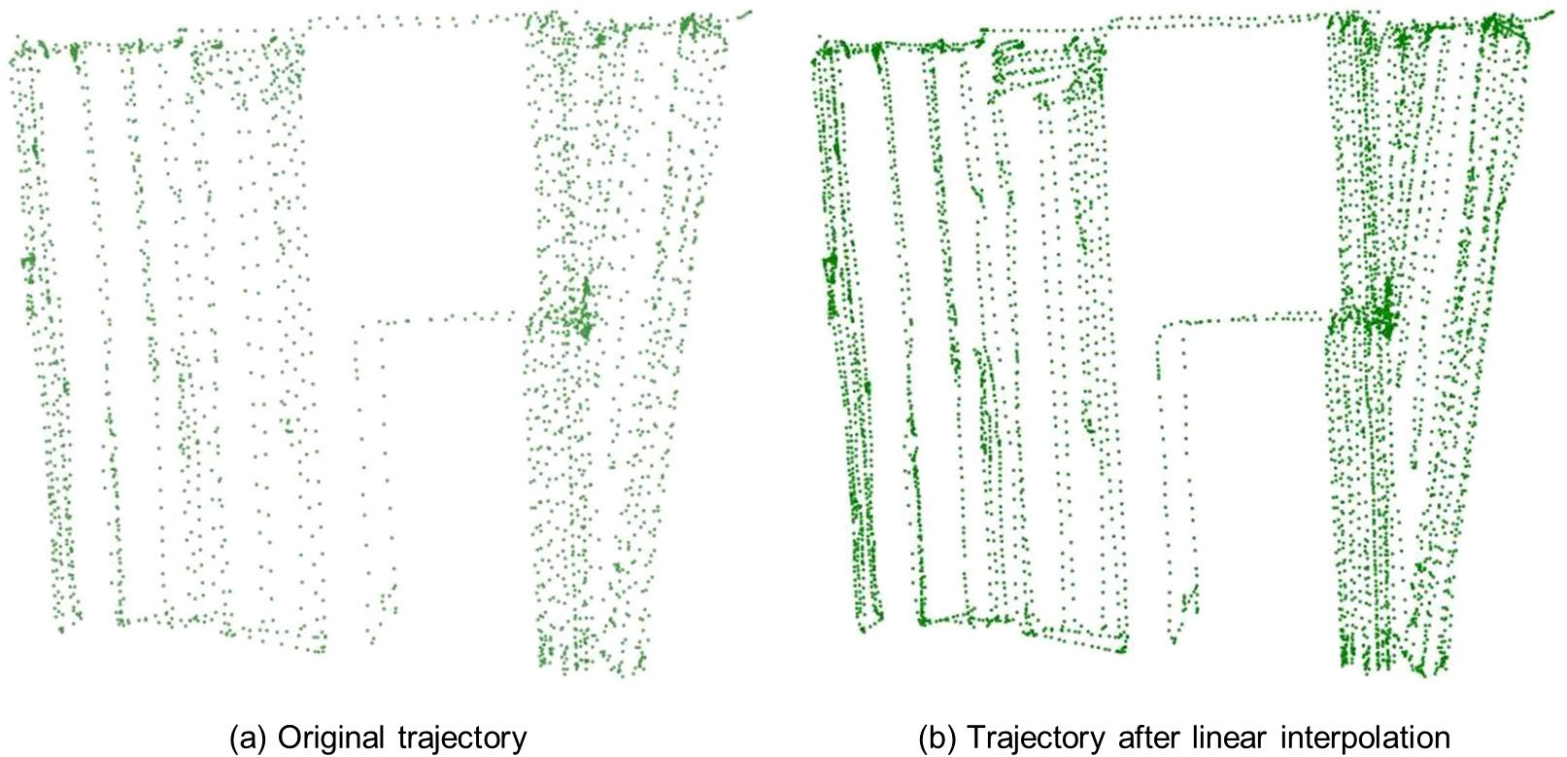
Results of linear interpolation.

For temporal continuity restoration, when the time interval between two consecutive points exceeds the normal sampling interval but does not reach the threshold for stopping or task switching, linear interpolation is used to recover intermediate points, thereby mitigating the effect of irregular sampling on trajectory segmentation and image modeling, as shown in **Figure 7**. Let the time difference between two adjacent points ${\tilde{p}}_{\text{i}}$ and ${\tilde{p}}_{\text{i}+1}$ be $\Delta {\text{t}}_{\text{i}}={\text{t}}_{\text{i}+1}-{\text{t}}_{\text{i}}$, when $\Delta {\text{t}}_{\text{s}}<\Delta {\text{t}}_{\text{i}}<\Delta {\text{t}}_{\text{stop}}$, where $\Delta {\text{t}}_{\text{s}}$ denotes the normal sampling interval and $\Delta {\text{t}}_{\text{stop}}$ denotes the stopping threshold, the intermediate point at time t is restored by linear interpolation according to the sampling frequency as shown in Equation 7:

(7) $$\tilde{p}\left(t\right)={\tilde{p}}_{i}+\frac{t-t_{i}}{t_{i+1}-t_{i}}({\tilde{p}}_{i+1}-{\tilde{p}}_{i}),\;t_{i}<t<t_{i+1}$$

#### 2.3.2 Candidate operation trajectory grouping and screening

After obtaining the regularized trajectory sequence $\tau^{\left(0\right)}$, candidate operation trajectories are grouped and screened according to the spatiotemporal continuity and motion patterns of the trajectories. This step removes evidently non-operational trajectories in advance, while also reducing the spatial span of subsequent trajectory images, mitigating pixel-level overlap between field trajectories and road trajectories during coordinate mapping, and improving the representation of local morphology, boundary structure, and motion patterns in the semantic segmentation stage. Based on existing trajectory identification schemes and the characteristics of practical agricultural machinery operation scenarios, this stage primarily removes long-distance reciprocating road trajectories, one-way road trajectories, and bypassing trajectories along field boundaries, while retaining typical field operation trajectories such as shuttle-pattern, set-pattern, and circumferential-pattern trajectories, as well as complex transitional trajectories including drifting trajectories, road-transfer trajectories, and field-road intersection trajectories, which are used as the input for subsequent segmentation.

To characterize the local spatiotemporal relationship between adjacent trajectory points, the Euclidean distance between two consecutive points is defined as Equation 8:

(8) $$d_{i}={\left\|\left(x_{i+1},y_{i+1}\right)-\left(x_{i},y_{i}\right)\right\|}_{2}$$

The heading variation between two consecutive points is defined as Equation 9:

(9) $$\Delta \theta_{i}=\min\left(\left|\theta_{i+1}-\theta_{i}\right|,360^{{}^{\circ}}-\left|\theta_{i+1}-\theta_{i}\right|\right)$$

and the local acceleration is defined as Equation 10:

(10) $$a_{i}=\frac{v_{i+1}-v_{i}}{t_{i+1}-t_{i}}$$

In addition, to characterize the degree of local spatial aggregation of trajectories, the point density within a neighborhood of radius ϵ is defined as Equation 11:

(11) $$p_{i}=\frac{\left|N_{\epsilon }\left({\tilde{p}}_{i}\right)\right|}{\pi \epsilon^{2}}$$

where $N_{\epsilon }\left({\tilde{p}}_{i}\right)$ denotes the set of neighboring points centered at ${\tilde{p}}_{i}$ within a radius of *ϵ*. These features characterize trajectory pattern differences from four aspects: spatial continuity, heading variation, motion state, and local aggregation. Road-transfer trajectories are typically characterized by large point spacing, small heading variation, low local density, and long-distance monotonic extension, whereas field-operation trajectories are more likely to exhibit relatively high local density, pronounced U-turns and heading changes, and reciprocating clustered distributions within the field. Accordingly, a candidate operation area extraction operator is constructed, and the resulting candidate trajectory set can be expressed as Equation 12:

(12) $$\tau_{c}={\text{Φ}}_{cand}\left(\tau^{\left(0\right)}\right)$$

To prevent points that are far apart from being incorrectly connected into the same trajectory segment due to GNSS drift or short-term signal interruptions, spatial distance-constrained segmentation is introduced after candidate area extraction. Given a distance threshold ${\text{D}}_{\text{t}}^{\left(\text{m}\right)}$, associated with the machinery type, the trajectory segmentation rule is defined as Equation 13:

(13) $$\left\{ \begin{array}{l} d_{i}\leq D_{t}^{\left(m\right)}\;\text{, }{\tilde{p}}_{i},{\tilde{p}}_{i+1}\text{ belong to same trajectory}\\ d_{i}>D_{t}^{\left(m\right)}\text{, new trajectory} \end{array}\right.$$

After this process, a set of candidate trajectory segments with group labels, ${\left\{L_{j}\right\}}_{j=1}^{M}$ is obtained, and the distance between any two adjacent trajectory points within each segment does not exceed the prescribed threshold.

The thresholds used in preprocessing and candidate trajectory grouping were determined according to the sampling characteristics of the onboard terminals, the expected working speeds of different machinery types, and preliminary tests on the IAEMP dataset. The temporal threshold was used to distinguish short-term sampling gaps from task interruptions or stopping states. The maximum reasonable speed threshold was set by machinery type to remove overspeed points that were physically inconsistent with field operation. The neighborhood radius and spatial distance threshold were used to constrain local point aggregation and prevent distant trajectory segments from being incorrectly connected after GNSS drift or communication interruption. These parameters were not used to directly determine the final operation area; instead, they served as conservative screening constraints before semantic segmentation. Therefore, uncertain mixed regions were retained for the subsequent CBGAM U-Net refinement rather than being removed only by hard thresholds.

#### 2.3.3 Trajectory image construction and label generation

**Figure 8:**
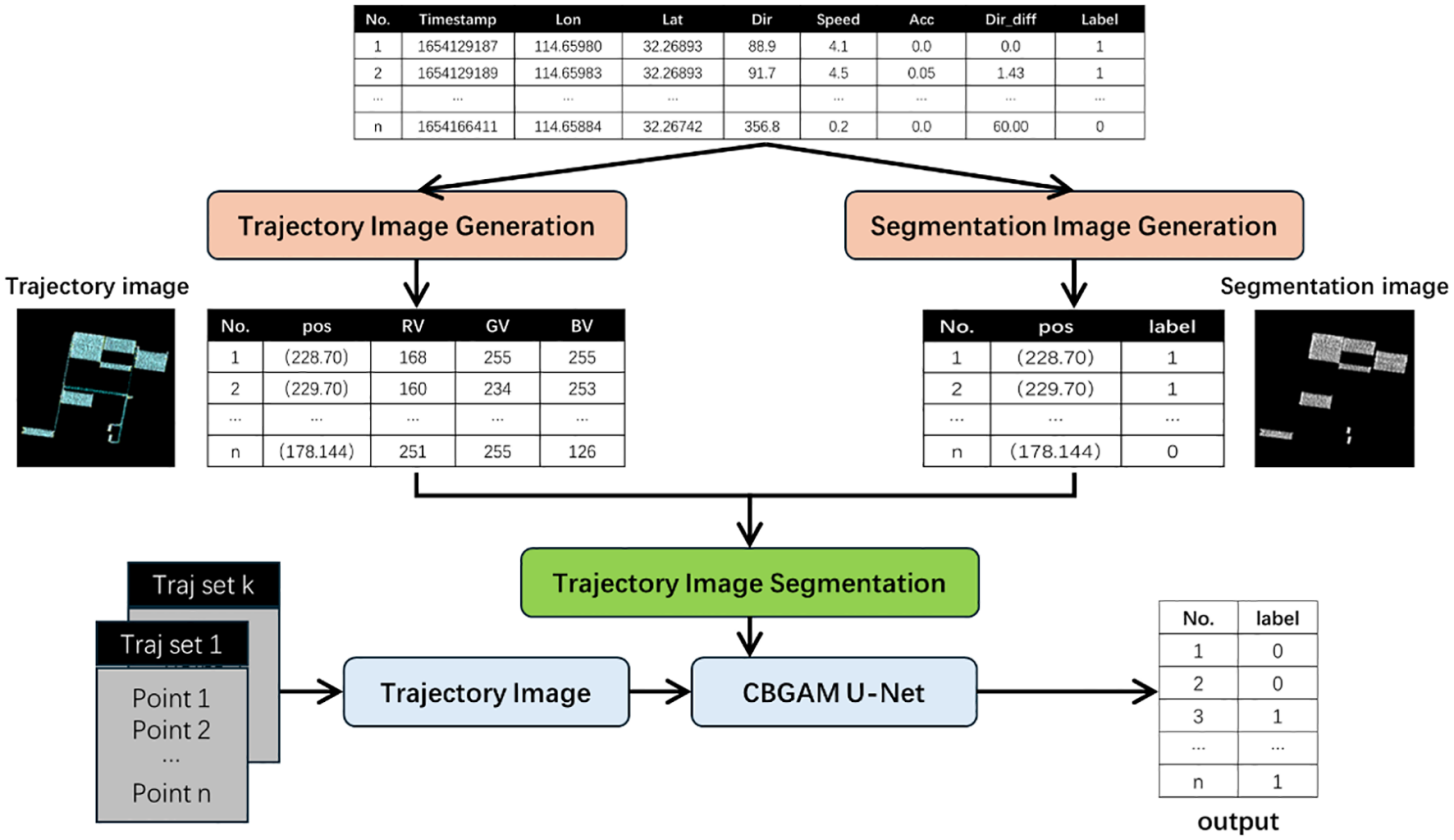
Trajectory image construction and labelling.

Candidate extraction based on spatiotemporal constraints can filter out a large number of obviously non-operational trajectories. However, for complex transitional scenarios such as field-road intersections, road turns, and drifting trajectories, stable and accurate classification remains difficult when relying solely on threshold-based rules. To further exploit the overall spatial morphology of trajectories and local motion variations, the candidate trajectory segments are mapped into multi-channel trajectory images, and corresponding label maps are constructed, thereby formulating agricultural machinery operation trajectory identification as a pixel-wise semantic segmentation task, as shown in **Figure 8**. Raw trajectory sequences can preserve point-level temporal relationships effectively, but they are not well suited to directly represent the overall spatial structure of field contours, local boundaries, and complex mixed trajectories. In contrast, trajectory images can encode position distribution, morphological boundaries, and local motion attributes within a unified representation space, thus providing an appropriate input form for subsequent segmentation models.

For each candidate trajectory group, the planar coordinates were first normalized within the spatial extent of the group and then discretized into a fixed-size image grid. Each trajectory point was projected onto the corresponding pixel according to its planar coordinates. When multiple trajectory points were mapped to the same pixel, their feature values were averaged to reduce the influence of local sampling-density variation. A three-channel trajectory image was subsequently generated. The first channel encoded normalized travelling speed, which helps distinguish field operation from road transfer and static drift. The second channel encoded normalized local acceleration, reflecting short-term motion-state transitions and local speed fluctuations. The third channel encoded normalized heading variation, which captures U-turns, headland movements, road turns, and reciprocating operation patterns. In this way, the generated trajectory images preserve both spatial morphology and local kinematic characteristics for semantic segmentation.

The corresponding label images were generated using the same coordinate-to-pixel mapping strategy. Pixels occupied by valid field-operation trajectories were assigned a label value of 1, whereas pixels corresponding to road-transfer, drifting, parking, or other non-operational trajectories were assigned a label value of 0. The resulting image-label pairs therefore constituted supervised samples for semantic segmentation training.

Let the candidate trajectory set T_c_ be transformed into a multi-channel image after coordinate discretization and attribute mapping, which is expressed as Equation 14:

(14) $$I=g\left(\tau_{c}\right)\in {\mathbb{R}}^{H\times W\times C}$$

Where H and W denote the image height and width, respectively, and C denotes the number of feature channels. The trajectory image is represented at a unified resolution, and the trajectory points within the same trajectory group are projected onto the image plane through a mapping from positional coordinates to pixel coordinates. A three-channel representation is adopted as Equation 15 bellow, in which the first, second, and third channels correspond to the normalized mappings of speed, acceleration, and heading variation, respectively.

(15) $$\left\{ \begin{array}{l} I^{\left(1\right)}(u,v)=Norm(v_{i})\\ I^{\left(2\right)}(u,v)=Norm(a_{i})\\ I^{\left(3\right)}(u,v)=Norm(\Delta \theta_{i}) \end{array}\right.$$

Where (*u*,*v*) denotes the image pixel coordinates, and *Norm*(·) denotes the normalization operation. The trajectory image represents the spatial structure of trajectories through pixel locations, while encoding the local motion features of trajectory points through pixel values, thereby enabling a joint representation of spatial and motion information. Correspondingly, a label image can be constructed according to the annotations, which is defined as Equation 16:

(16) Y(u,v)∈{0,1}

**Figure 9:**
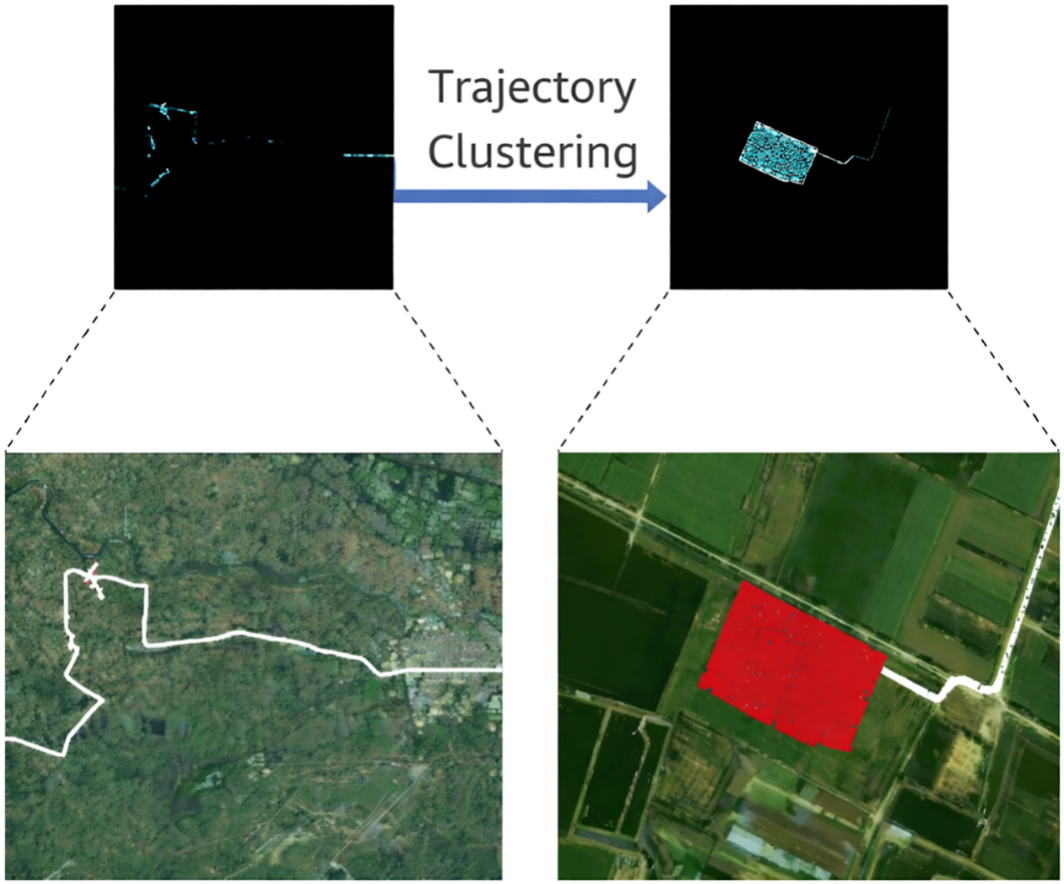
Trajectory image generation.

Where *Y*(*u*,*v*) = 1 indicates a pixel corresponding to a field-operation trajectory, and *Y*(*u*,*v*) = 0 indicates a pixel corresponding to a road or other non-operational trajectory. The label image shares the same coordinate mapping relationship as the trajectory image, thereby forming a one-to-one supervised sample pair, as shown in **Figure 9**.

#### 2.3.4 Semantic segmentation-based refinement

After the trajectory image I and the label image Y are constructed, a semantic segmentation network is introduced to establish the mapping from the trajectory image to the segmentation output as shown in Equation 17:

(17) $$\hat{Y}=f_{\text{Θ}}(I)$$

**Figure 10:**
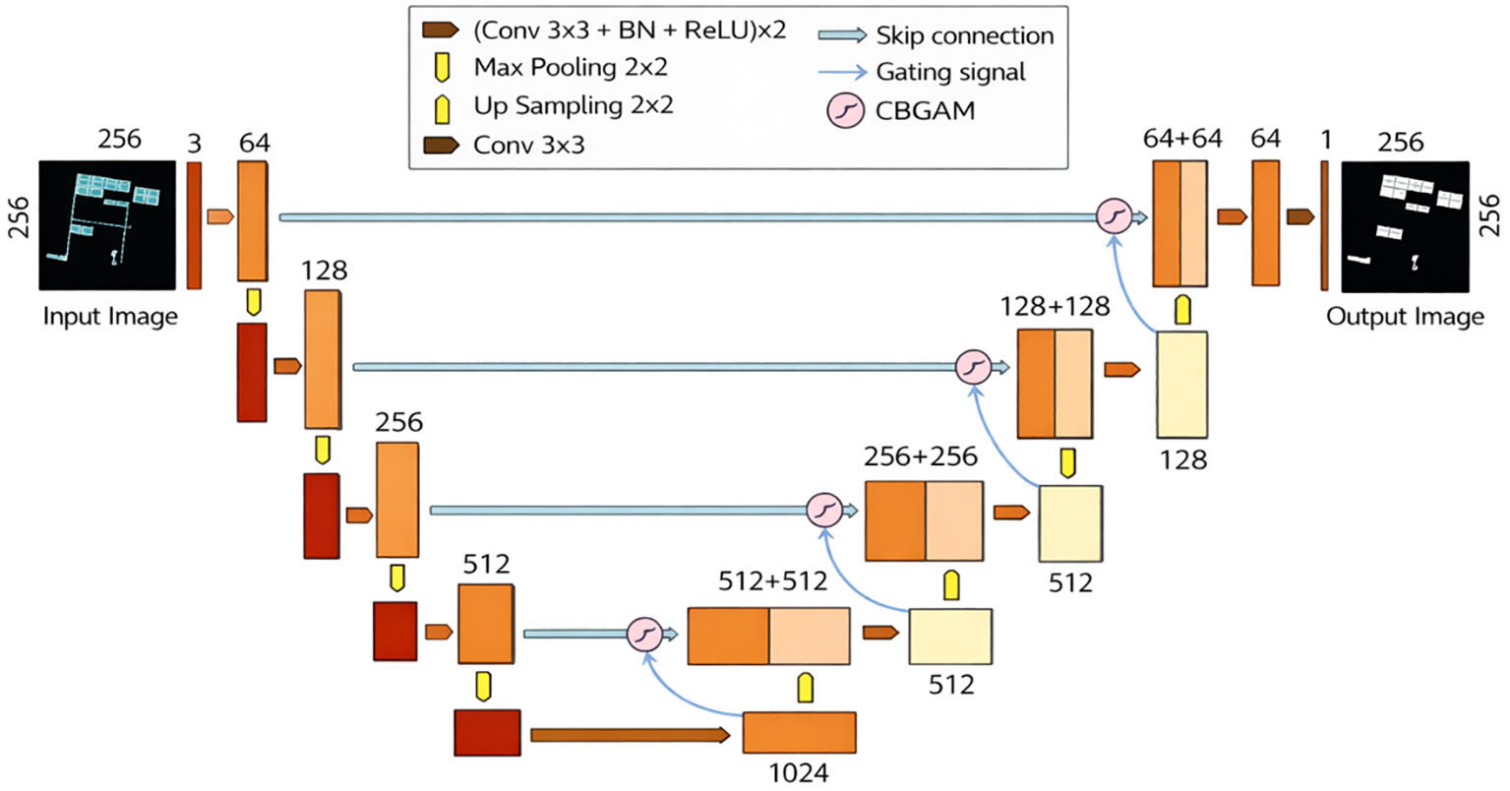
Structure of CBGAM U-Net.

Where $\hat{Y}$ denotes the pixel-wise probability map predicted by the model. The input to the semantic segmentation model consists of the candidate trajectory images obtained after grouping and screening in the previous stage. Its purpose is to further discriminate complex mixed regions, including r road-transfer trajectories adjacent to field parcels, drifting trajectories, field-road intersections, and road-transfer trajectories, thereby yielding clearer boundaries of valid operation trajectories. Based on the original U-Net architecture, an attention mechanism was incorporated and a weighted focal loss function was adopted for training. The resulting improved semantic segmentation model is termed CBGAM U-Net, and its architecture is shown in **Figure 10**.

**Figure 11:**
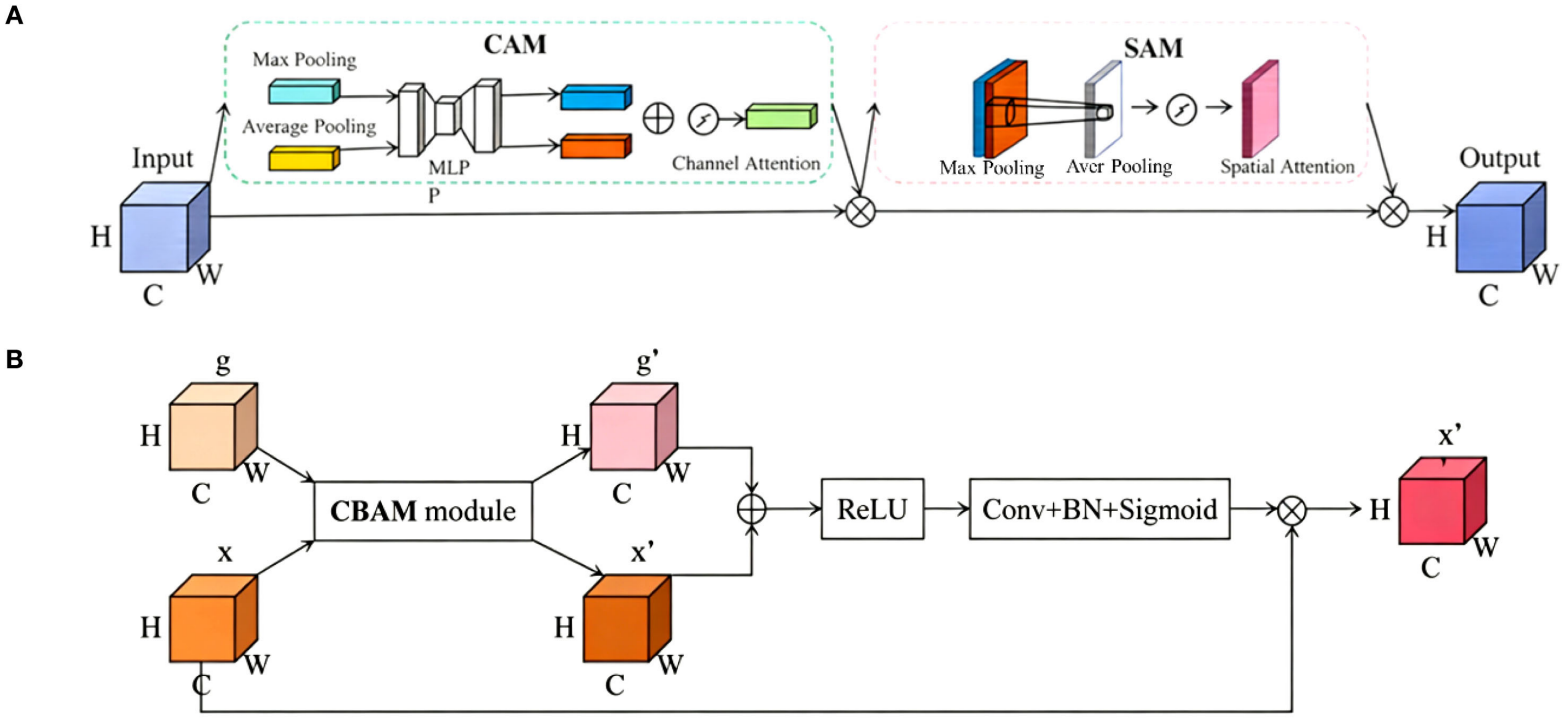
Structure of attention module. **(A)** CBAM, **(B)** CBGAM.

The Convolutional Block Attention Module (CBAM) (Woo et al., 2018) is a general and lightweight module that relies only on simple convolution and pooling operations and therefore incurs low computational overhead. By extracting channel-wise and spatial information from the input features to generate weighting matrices, CBAM enables the network to focus more strongly on target regions, thereby facilitating the simultaneous extraction of both global and local trajectory features. In addition, following the idea proposed by Oktay (Oktay et al., 2018) integrating an attention mechanism into the skip-connection stage not only helps preserve information from the original image but also allows the corresponding weights to be learned by the network. Accordingly, CBAM is incorporated into the skip-connection stage in this study, as shown in **Figure 11a**, and a Convolutional Block and Gated Attention Module (CBGAM) is proposed. The schematic of CBGAM is shown in **Figure 11b**, where *g* denotes the feature map generated in the downsampling stage and *x* denotes the feature map generated in the upsampling stage.

The CBGAM module jointly enhances feature maps by cascading channel attention and spatial attention. In the channel attention branch, global max pooling and global average pooling are first applied to the input feature map, and the resulting descriptors are passed through a shared multilayer perceptron and activation function to generate channel-wise weights, thereby emphasizing informative channels. In the spatial attention branch, the output of the previous step is taken as input; max pooling and average pooling are performed along the channel dimension, followed by convolution and a sigmoid function to generate spatial weights, thereby highlighting informative spatial regions. Based on this design, CBGAM U-Net performs channel and spatial attention calculations simultaneously on the downsampled and upsampled feature maps at the corresponding encoder and decoder layers. The enhanced features are then concatenated with the downsampled feature maps and fed into the decoder, thereby improving the representation of critical regions in agricultural machinery trajectory images. Combined with a pixel-wise classification strategy, each pixel is finally classified as either an agricultural machinery trajectory pixel or a road trajectory pixel, enabling fine-grained trajectory identification based on semantic segmentation.

Compared with conventional channel-only or spatial-only attention mechanisms, the proposed CBGAM module jointly models channel importance and spatial response under trajectory-image representations, thereby enhancing the discrimination of elongated operation trajectories, sparse drifting trajectories, and mixed field-road boundary regions. To evaluate the effectiveness of the introduced attention mechanism, ablation experiments and comparative analyses with other mainstream attention modules will be further conducted.

### 2.4 Vector buffer-based operation area estimation

**Figure 12:**
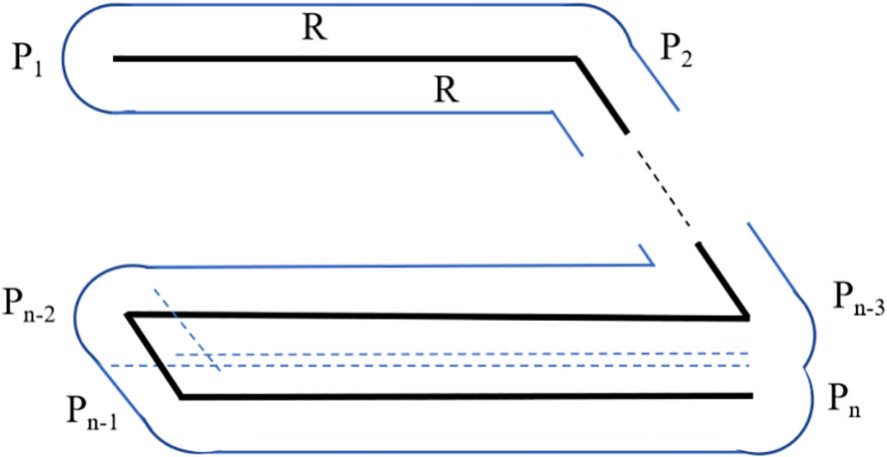
Schematic diagram of vector buffer algorithm.

After obtaining the valid operation trajectory set τ_op_, operation area estimation is performed using a spatial analysis method based on vector buffers. The machinery operation process can be regarded as the continuous movement of an implement along the operation trajectory while covering a spatial region with a certain working width. Accordingly, the valid operation trajectories can be approximated as the centerlines of the implement-covered region, and the final operation area can be reconstructed through width compensation and geometric merging, as shown in **Figure 12**.

From a geometric perspective, let the valid operation trajectory set be denoted as ${\text{τ}}_{\text{op}}={\left\{{\text{L}}_{\text{j}}\right\}}_{j=1}^{\text{M}}$. The real operation coverage region A^*^ can then be approximated as the Minkowski sum of the trajectory centerlines and the implement footprint set, as shown in Equation 18:

(18) $$A^{*}\approx \overset{M}{\underset{j=1}{\cup }}\left(L_{j}⊕S_{W}\right)$$

Where ⊕ denotes the Minkowski sum, and S_W_ denotes the lateral footprint set associated with the implement working width. By approximating S_W_ as a closed disk D(r) with radius r, the coverage recovery problem is transformed into the construction of buffers around linear features. Under this formulation, agricultural machinery operation area estimation can be uniformly described as a geometric recovery process consisting of centerline reconstruction, region expansion, boundary correction, and area calculation.

#### 2.4.1 Width-compensated vector buffering

Before vector-buffer construction, cubic spline interpolation was introduced to reconstruct smoother operation centerlines from discrete trajectory points. Raw GNSS trajectories are recorded as polylines composed of discrete points. When the sampling interval is irregular or when local positioning drift occurs, directly buffering such broken or angular polylines may produce local boundary spikes, artificial gaps, or excessive outward expansion near turns and endpoints. Cubic spline interpolation provides a smooth and continuous curve representation with continuous first- and second-order derivatives, which is suitable for approximating the physical movement of agricultural machinery during continuous field operation. Compared with simple linear interpolation, cubic spline interpolation better preserves the continuity of operation passes and reduces abrupt direction changes caused by sparse sampling or positioning jitter. This smoothing process does not change the basic spatial trend of the operation trajectory; rather, it improves the geometric stability of the subsequent buffer operation and reduces local area errors caused by polyline artifacts.

For each continuous trajectory segment L_j_, a vector buffer is used to transform the line-based centerline into an area-based operation coverage region. Let W_j_ denote the nominal working width of the implement corresponding to trajectory segment L_j_. The buffer radius w_j_ is then defined as Equation 19:

(19) $$w_{j}=\frac{W_{j}}{2}+\delta$$

Where, $\frac{{\text{W}}_{\text{j}}}{2}$ represents half of the implement working width. The compensation term *δ* was introduced to account for the mismatch between the recorded GNSS trajectory and the actual implement coverage centerline. This mismatch may be caused by GNSS positioning bias, non-central installation of the onboard terminal, implement offset relative to the tractor or harvester body, local pass-spacing variation, and slight deviations during headland turning. If only half of the nominal implement width is used as the buffer radius, the recovered coverage region may contain artificial gaps between adjacent operation passes, especially when the GNSS trajectory is shifted from the true operation centerline. In this study, *δ* was set to 1.5 m according to the terminal positioning characteristics, implement widths, and preliminary comparison between buffer results and manually checked operation boundaries. To avoid systematic overestimation caused by this outward compensation, the same scale was subsequently used for inward boundary correction after polygon union. Thus, the compensation and inward correction were jointly used to balance coverage completeness and boundary accuracy.

Accordingly, the buffer corresponding to the *j*-th trajectory segment can be expressed as Equation 20:

(20) $$B_{j}=B\left(L_{j},w_{j}\right)$$

Under a morphological representation, this can be written as Equation 21:

(21) $$B_{j}=L_{j}⊕D\left(w_{j}\right)$$

#### 2.4.2 Polygon union and boundary correction

This operation corresponds to a morphological dilation of the trajectory centerline and is used to simulate the actual operation coverage on both sides of the trajectory. Let the set of buffers generated for all trajectory segments be denoted as Equation 22:

(22) $$\beta ={\left\{B_{j}\right\}}_{j=1}^{M}$$

Then the initial operation region A_0_ can be expressed as Equation 23:

(23) $$A_{0}=\overset{M}{\underset{j=1}{\cup }}B_{j}$$

Through the union operation, overlapping regions between adjacent operation passes are not counted repeatedly, and locally discrete buffers can be merged into a continuous operation area. However, the buffering operation itself introduces an outward expansion effect near field boundaries, turning regions, and trajectory endpoints, which may lead to systematic overestimation. To reduce this bias, a boundary inward correction is further applied to the initial operation region A_0_ after the union. If the same correction scale δ, as that used in buffer compensation is adopted, the final operation region can be expressed as Equation 24:

(24) $$A=\text{Buffer}\left(A_{0},-\delta \right)$$

Under a morphological representation, this can be written as Equation 25:

(25) $$A=A_{0}⊖D\left(\delta \right)$$

Where ⊖ denotes the Minkowski difference and can also be interpreted as a morphological erosion applied to the initial operation region. This operation is used to suppress area overestimation caused by boundary expansion and excessive compensation, thereby balancing coverage completeness and boundary accuracy in the recovered operation region. Finally, the operation area S is directly calculated from the corrected operation region A in the planar projected coordinate system.

### 2.5 Experimental design and validation

To systematically evaluate the effectiveness and reliability of the proposed method in real-world agricultural machinery operation management scenarios, a two-level experimental framework was established, consisting of platform-level validation and field validation with RTK ground truth. The platform-level validation targets practical business environments and focuses on the capability of the proposed method in operation trajectory identification, the consistency of operation area estimation, and the deployment performance on the cloud platform under complex historical operation records. In contrast, the RTK-based field validation relies on an independent high-precision reference and focuses on assessing the geometric fidelity of the recovered operation areas.

#### 2.5.1 Platform-level validation

For platform-level validation, historical operation records from the Smart Agricultural Machinery Management Platform were used to evaluate the proposed method under practical cloud-platform business conditions. The validation samples were selected from the IAEMP historical trajectory records after removing records with missing terminal IDs, incomplete time information, abnormal coordinate ranges, unavailable implement width, or extremely short operation duration. Duplicate records generated by repeated upload of the same task were also removed. From the remaining valid historical records, 1,000 samples were selected to cover the main interference scenarios encountered in practical operation management, including regular field parcels, parcels containing road-transfer trajectories during operation, static or drifting trajectories, transfer trajectories close to headlands, parcels with relatively few reciprocating passes, and sparse-trajectory parcels. These samples were used to evaluate trajectory identification robustness, area estimation consistency, and cloud-platform processing efficiency.

**Figure 13:**
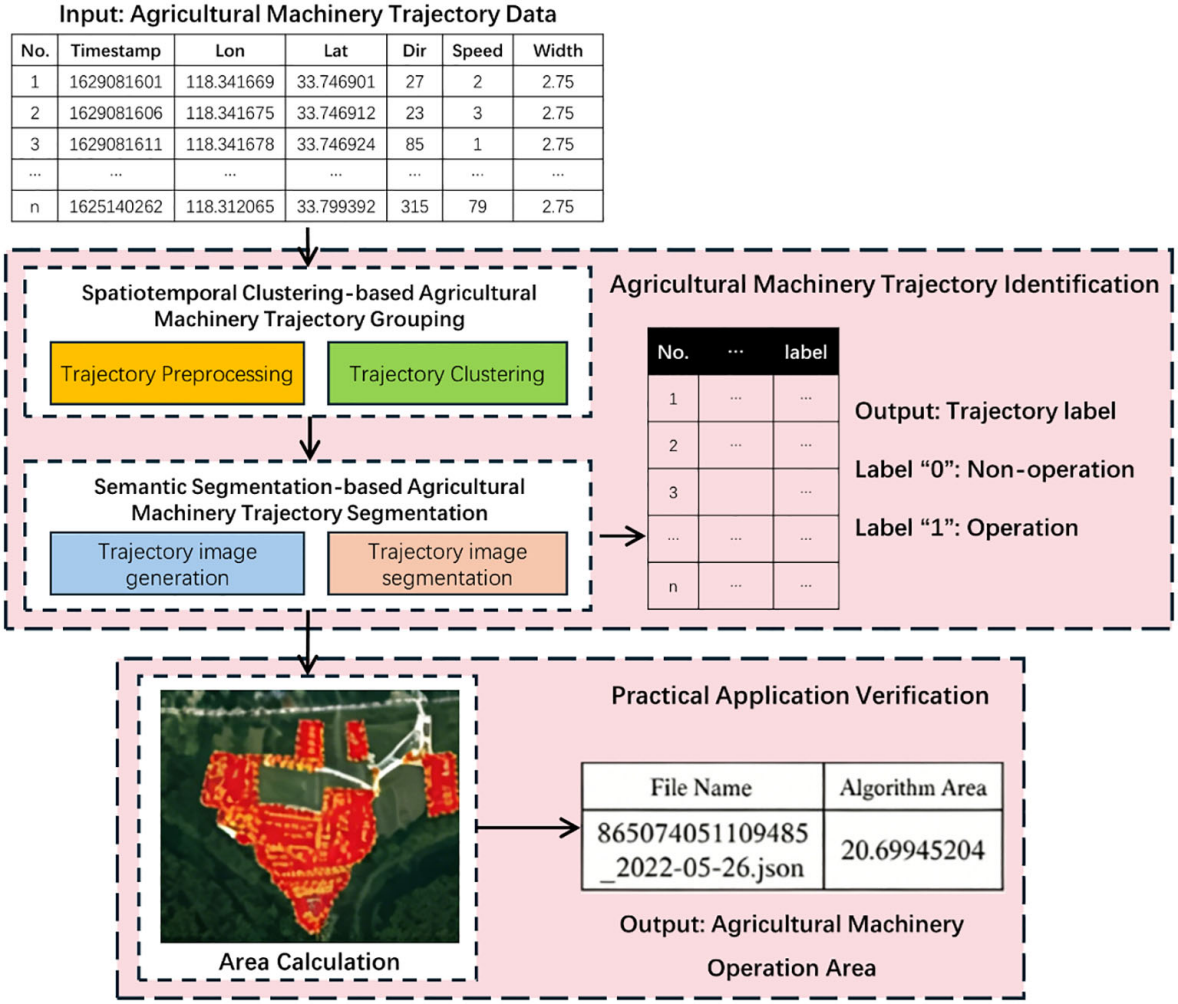
Platform-level validation method.

For each sample, the complete processing chain was executed, including trajectory preprocessing, candidate operation trajectory grouping, trajectory image construction, semantic-segmentation-based refinement, vector-buffer-based area estimation, and visualization output. **Figure 13** summarizes the platform-level validation workflow. This validation was designed to test the deployability and stability of the method on heterogeneous historical platform data. It should be distinguished from the RTK-based field validation, which was conducted independently to assess geometric area accuracy using high-precision reference measurements.

#### 2.5.2 Field validation with RTK ground truth

To further evaluate the geometric validity of the estimated operation area, field validation was conducted using Real-Time Kinematic (RTK) positioning measurements as an independent ground-truth reference. RTK positioning provides higher positioning accuracy than ordinary GNSS trajectory records collected by onboard operation terminals and is therefore suitable for measuring field boundaries and validating area estimation results. In the field experiment, the actual parcel boundary was first surveyed using RTK equipment. For parcels containing obstacles or non-operational regions, the corresponding internal non-operational boundaries were also measured and subtracted from the total parcel area to obtain the ground-truth operation area. The agricultural machine then performed the actual operation within the plot, during which the onboard terminal recorded the operation trajectory, terminal ID, implement width, operation type, and start and end times.

The proposed method was applied only to the onboard terminal trajectory to generate the estimated operation boundary and operation area. The RTK-measured area was not used during model training or algorithm processing, but only as an independent reference for final validation. Let the estimated area be S*_EST_* and the RTK-measured ground-truth area be S*_RTK_*. The operation area accuracy was calculated as follows (Equation 26):

(26) $$Acc_{S}=\left(1-\frac{\left|S_{EST}-S_{RTK}\right|}{S_{RTK}}\right)\times 100\%$$

#### 2.5.3 Model training settings and evaluation metrics

All segmentation models were trained and tested using the same trajectory-image dataset and the same data split to ensure a fair comparison. The dataset was divided into training, validation, and test sets at a ratio of 8:1:1, and trajectory images from the same original operation task were kept within the same subset to avoid data leakage. The input image size was set to 256 × 256 pixels. The models were implemented using PyTorch and trained on NVIDIA GeForce RTX 3060 Laptop GPU. The optimizer was Adam, with an initial learning rate of 0.001, batch size of 4, and training epochs of 20. The weighted focal loss was used to reduce the influence of class imbalance between operation and non-operation pixels.

The segmentation performance was evaluated using accuracy, precision, recall, F1-score. In the binary segmentation task, operation pixels were treated as positive samples and non-operation pixels as negative samples. These metrics jointly evaluate the overall classification correctness, the reliability of detected operation pixels, the completeness of operation trajectory extraction, and the degree of overlap between predicted and labeled operation regions.

## 3 Results and discussion

### 3.1 Operation trajectory identification

The trajectory identification performance of the proposed method was evaluated on the IAEMP test set using the experimental design described in Section 2.5.3. FCN, SegNet, U-Net, Deeplab V3p, and the proposed CBGAM U-Net were compared under the same trajectory-image construction scheme and the same training/testing split. The comparison focused on both segmentation accuracy and inference efficiency, because the target application is cloud-platform deployment for large-scale agricultural machinery operation records.

**Figure 14:**
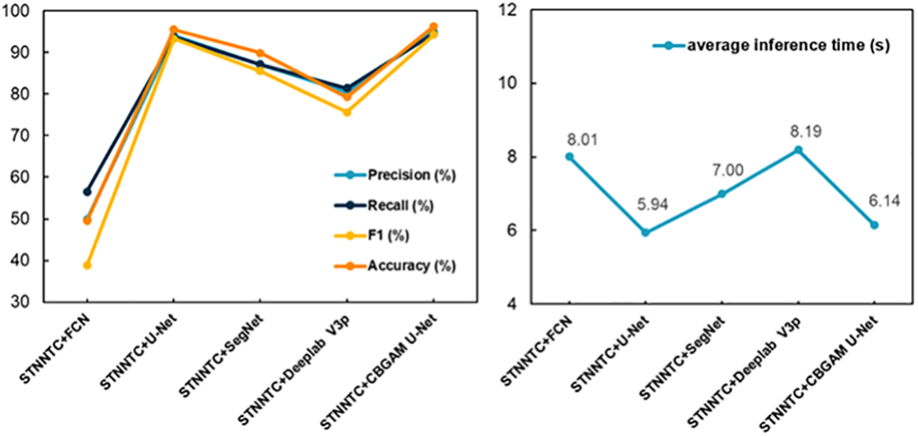
Model performance comparison.

The comparative results are presented in **Figure 14**. Among the backbone models, U-Net achieved the best overall performance relative to FCN, SegNet, and Deeplab V3p, ranking first in both F1-score and accuracy, with values of 93.29% and 95.54%, respectively. It also yielded the shortest average inference time, at 5.94 s. These results indicate that the skip connections in U-Net effectively preserve deep features, while the combination of deconvolution and upsampling facilitates image reconstruction and alleviates the loss of pixel-level spatial information caused by pooling operations. Compared with U-Net, CBGAM U-Net further improved the F1-score and accuracy by 1.00% and 0.78%, respectively, while increasing the average inference time by only 0.20 s. This improvement suggests that the introduced attention mechanism enhances the extraction of both channel-wise and spatial features from trajectory images, thereby improving identification accuracy with only a marginal increase in computational cost.

To evaluate the individual contributions of different components in the proposed CBGAM U-Net, five ablation experiments were conducted. Specifically, the effects of removing the channel-attention branch, removing the spatial-attention branch, removing the gated-attention branch, replacing the weighted focal loss (FL) with binary cross-entropy loss (BCE Loss, BL), and substituting the proposed CBGAM module with Attention U-Net were investigated. The corresponding results are presented in **Table 2**, where the bold values indicate the best performance.

**Table 2:** Attention performance comparison.

Model variant	Precision (%)	Recall (%)	F1-score (%)	Accuracy (%)	Inference time (s)
CBGAM U-Net (no CAM)	94.65	**94.67**	94.21	96.25	**5.97**
CBGAM U-Net (no SAM)	94.64	94.40	94.02	95.99	5.99
CBGAM U-Net (no GAM)	94.56	93.79	93.50	95.67	6.12
CBGAM U-Net + BCE loss	94.87	94.49	94.13	96.11	6.16
Attention U-Net	93.91	93.17	92.90	95.63	6.06
CBGAM U-Net	**95.07**	94.50	**94.29**	**96.32**	6.14

Bold ones are the best performance.

Compared with the complete CBGAM U-Net, all ablated variants showed performance degradation to different extents, indicating that each introduced component contributed positively to trajectory-image segmentation. Removing the channel-attention branch caused only a slight decrease, whereas removing the spatial-attention branch led to a larger reduction, suggesting that spatial feature enhancement is particularly important for distinguishing field boundaries and mixed trajectory regions. The most obvious degradation occurred after removing the gated-attention branch, with the F1-score and accuracy decreasing by 0.79% and 0.65%, respectively, indicating that the gated-attention mechanism improves the selective enhancement of critical trajectory features in complex field-road transition scenarios. Replacing weighted focal loss with BCE loss also reduced model performance, confirming that focal loss is more suitable for handling class imbalance and hard-sample learning in agricultural machinery trajectory segmentation. In addition, CBGAM U-Net outperformed Attention U-Net in Precision, Recall, F1-score, and Accuracy, further demonstrating the effectiveness of the proposed hybrid attention structure. Although the introduced modules slightly increased inference time, the additional computational cost remained limited, and the proposed model still achieved a practical balance between segmentation accuracy and engineering deployability.

**Figure 15:**
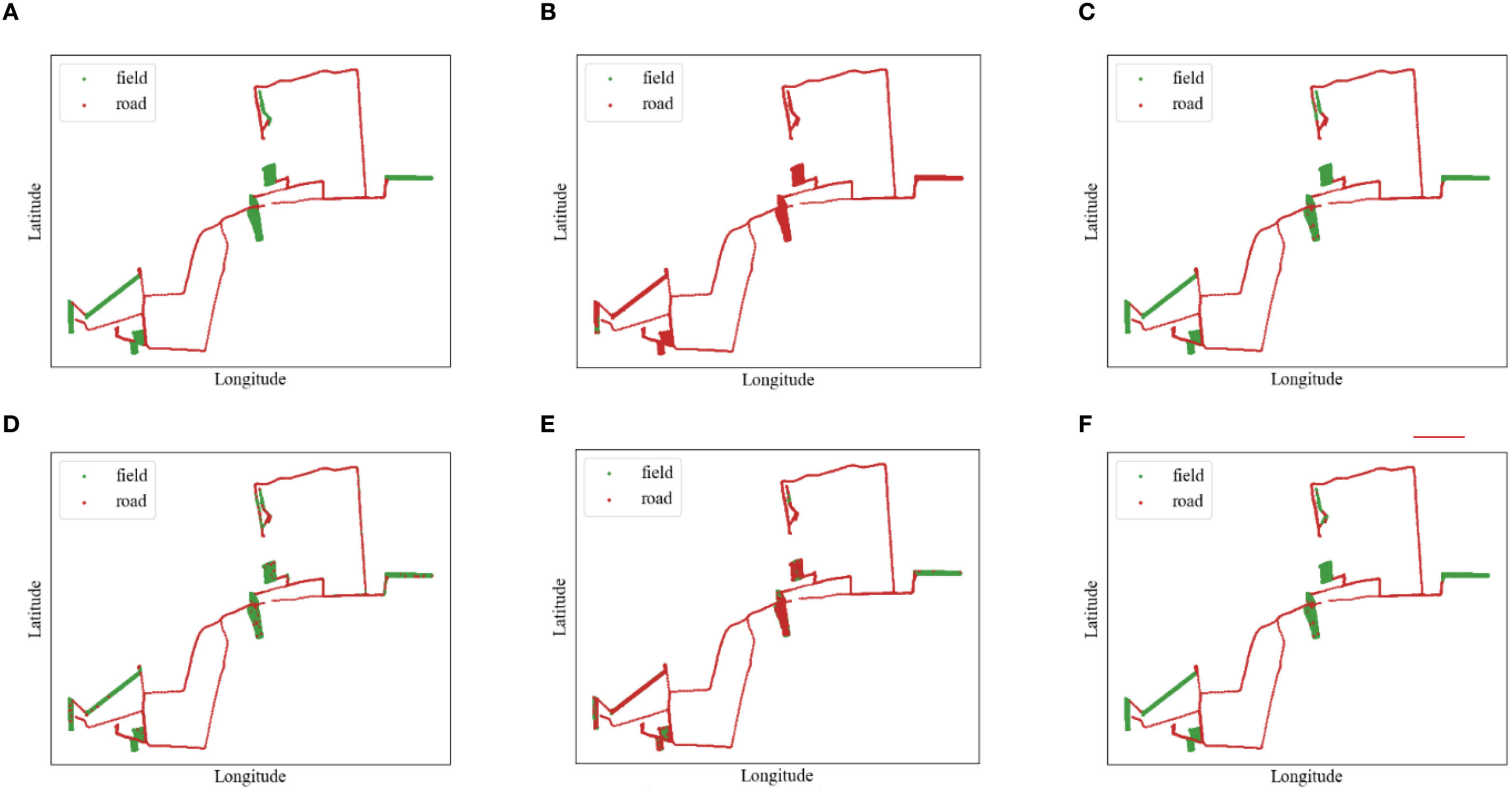
Visualization results of different backbone models. **(A)** ground truth, **(B)** STNNTC+FCN, **(C)** STNNTC+U-Net, **(D)** STNNTC-SegNet, **(E)** STNNTC+Deeplap C3p, **(F)** STNNTC+CBGAM U-Net.

As shown in **Figure 15**, the recognition results of the five methods on a representative trajectory sample exhibit clear difference. FCN showed the poorest performance, as its upsampling was insufficient to recover detailed structures. Deeplab V3p tended to lose local detail information under the selected dilation settings, resulting in blurred boundaries or missed detections for small-scale targets. SegNet was able to identify most agricultural machinery operation trajectories, but the loss of deep semantic information led to misclassification of road-transfer trajectories adjacent to field parcels and sparsely distributed field trajectories. U-Net, although more effective overall, lacked dynamic attention to critical regions and therefore still misidentified some road trajectories near field parcels as operation trajectories. In contrast, CBGAM U-Net achieved the best performance, effectively capturing global contextual information and improving the identification accuracy of road-transfer trajectories adjacent to field parcels.

### 3.2 Operation area estimation

**Figure 16:**
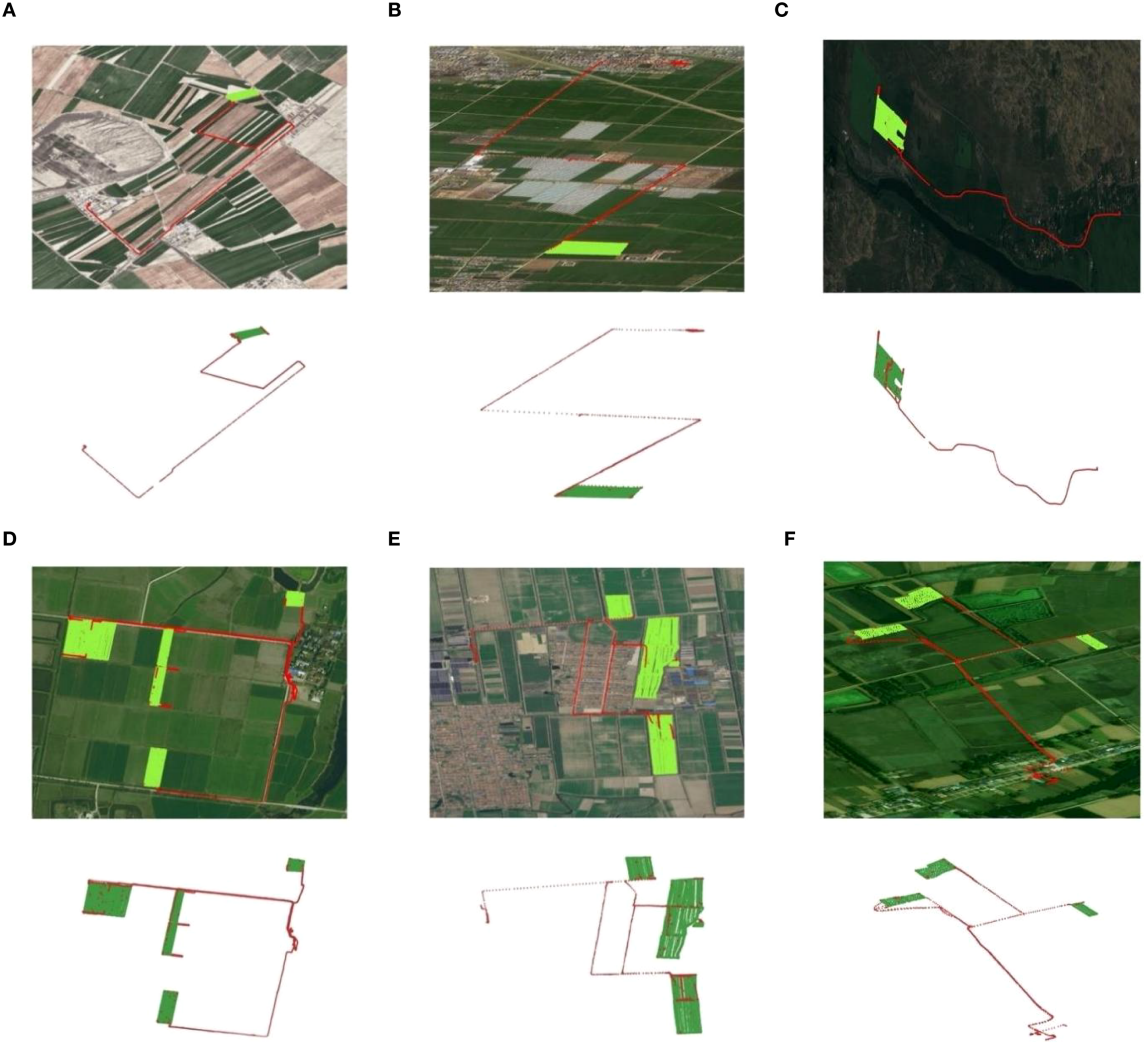
Operation areas. **(A)** Operation area 001, **(B)** Operation area 002, **(C)** Operation area 003, **(D)** Operation area 004, **(E)** Operation area 005, **(F)** Operation area 006.

Furthermore, six real agricultural machinery operation trajectories, comprising a total of 14 field parcels, were selected for testing. The proposed field–road trajectory classification method was applied, and the identification results are shown in **Figure 16**. Based on the identified field-operation trajectories for these 14 parcels, the previously described area estimation algorithm was used to calculate the operation areas. Among the selected trajectories, operation areas 001 to 003 each contained one parcel, operation area 004 contained five parcels, and operation areas 005 and 006 contained three parcels each. To evaluate the accuracy of the operation area estimation method presented in this chapter, the actual areas of the 14 field parcels associated with the six trajectories were measured and used as the ground-truth operation area.

The cubic-spline-interpolation-based buffer area algorithm was validated using agricultural machinery field-operation trajectory data from 14 parcels. As shown in **Table 3**, for typical agricultural machinery operation types, including rotary tillage, maize harvesting, rice transplanting, and rice harvesting, the absolute area estimation error of the algorithm remained below 3% in all cases. Specifically, the maximum error was 2.66% for maize harvesting (parcel 005_1), whereas the minimum error was 0.04% for rice harvesting (parcel 006_1). These results indicate that the proposed algorithm maintains stable estimation accuracy across different operation types and implement widths (1.9–4.0 m), demonstrating its applicability to operation area estimation under multiple agricultural operation scenarios.

**Table 3:** Operation area estimation results.

No.	Operation type	Width (m)	Sreal (m^2^)	Sest (m^2^)	Error (%)
001	Tillage	2.7	27048.27	26396.30	2.46
002	Tillage	2.7	27117.34	26973.41	0.53
003	Corn harvester	2.82	94307.45	94148.94	0.16
004_1	Transplanter	1.9	1441.05	1408.09	2.34
004_2	Transplanter	1.9	3478.11	3443.73	0.99
004_3	Transplanter	1.9	2236.77	2191.73	2.05
004_4	Transplanter	1.9	1971.58	1980.38	0.44
004_5	Transplanter	1.9	10079.44	9889.47	1.92
005_1	Corn harvester	4.0	77028.35	75032.49	2.66
005_2	Corn harvester	4.0	49386.85	48517.65	1.79
005_3	Corn harvester	4.0	19508.58	19107.94	2.09
006_1	Rice harvester	2.5	2167.31	2168.34	0.04
006_2	Rice harvester	2.5	1190.99	1216.60	2.10
006_3	Rice harvester	2.5	3208.05	3233.64	0.79

The cubic-spline-smoothed vector-buffer area estimation method was evaluated using agricultural machinery field-operation trajectories from 14 parcels. As shown in **Table 3**, the tested operation types included rotary tillage, corn harvesting, rice transplanting, and rice harvesting, with implement widths ranging from 1.9 to 4.0 m. The absolute area estimation errors of the 14 parcels were all below 3.00%, indicating that the proposed method maintained stable area estimation accuracy across different operation types and implement widths. These results suggest that combining refined operation trajectory extraction, trajectory smoothing, width-compensated buffering, and inward boundary correction can effectively reduce area bias caused by repeated operation, local missed operation, and positioning deviation.

### 3.3 Deployment performance on the cloud platform

**Figure 17:**
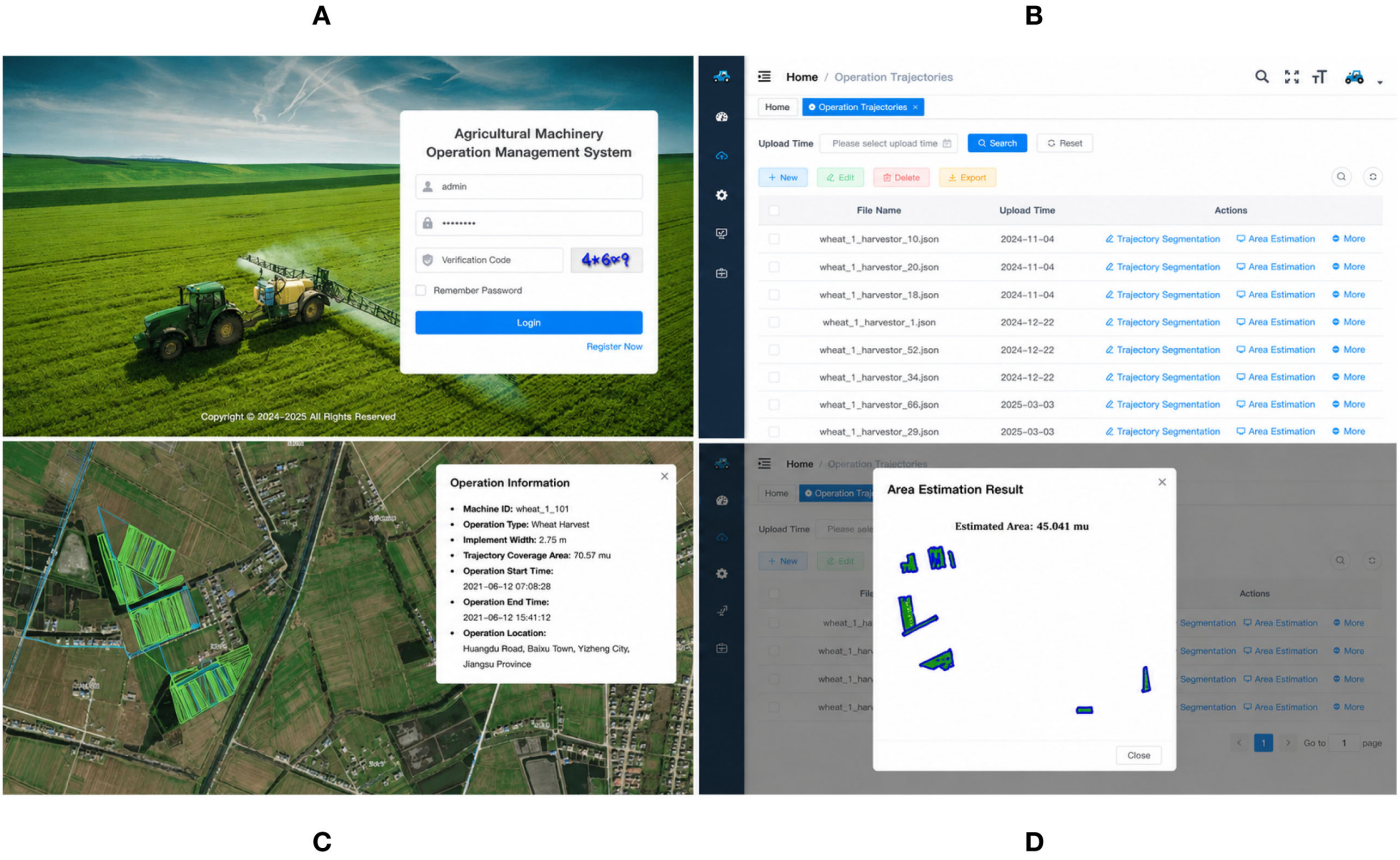
**(A)** Login interface, **(B)** Trajectory management interface, **(C)** Field-operation trajectory visualization interface, **(D)** Operation-area estimation result interface.Practical deployment of agricultural machinery management cloud platform.

The proposed trajectory identification and operation area estimation methods were integrated into the agricultural machinery cloud platform as a software package, as shown in **Figure 17**. The platform test evaluated the time consumption of trajectory data upload, field-road trajectory classification, area estimation, and visualization. As shown in **Table 4**, file upload, area calculation, and visualization required relatively short processing times, whereas the field-road trajectory classification step accounted for most of the total processing time, especially for complex or large-scale operation records. For example, File 4 required a longer classification time because it contained a larger operation area and more complex trajectory structure. Nevertheless, the system still achieved a processing efficiency of 5.08 mu/s for this file. Across the tested files, the platform maintained practical processing efficiency for cloud-based operation management. Here, 1 mu is equal to 666.67 m².

**Table 4:** Platform performance.

Item	File 1	File 2	File 3	File 4	File 5
Upload time (s)	1.51	1.32	1.33	2.24	1.22
Grouping time (s)	25.09	10.16	9.93	75.86	3.82
Calculate time (s)	2.12	1.31	1.14	5.25	0.91
Visualization time (s)	3.12	2.19	2.32	4.58	1.23
Time consume (s)	31.84	14.98	14.72	87.93	7.18
Area (mu)	96.20	34.53	39.20	447.46	22.36
Efficiency (mu/s)	3.02	2.30	2.66	5.08	3.11

The experimental results indicate that the system performs well when processing routine data, with the time consumption of file upload, area estimation, and visualization remaining within a reasonable range, demonstrating good stability and efficiency. However, when processing complex operation data or datasets of larger size, such as File 4 in the test, the time required for field–road trajectory classification increased substantially, leading to a longer overall processing time. Even under this condition, the system still achieved an operating efficiency of 5.08 mu/s, indicating its adaptability under different conditions and its relatively high computational efficiency.

**Figure 18:**
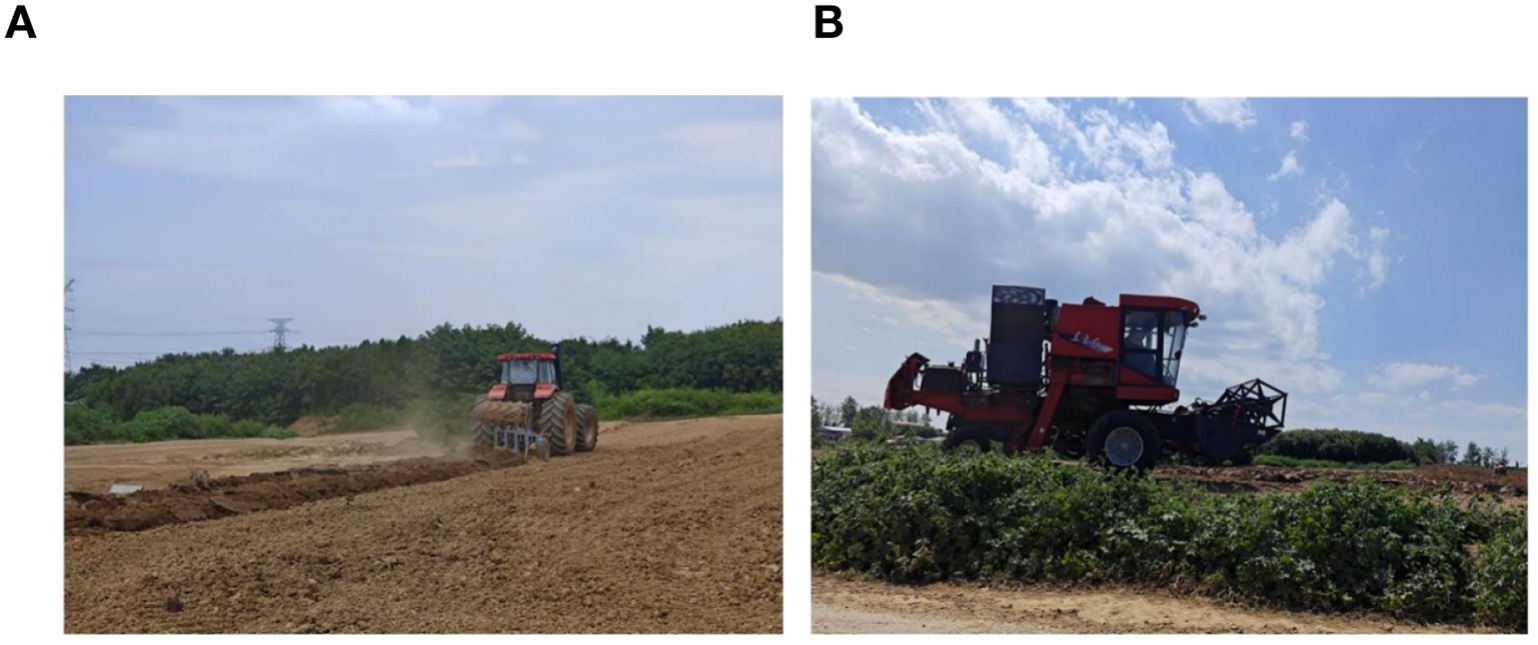
Operation type. **(A)** tractor, **(B)** harvester.

Then, two types of operations, namely wheat harvesting and rotary tillage, were conducted by machine operators in the experimental plots. After the operations were completed, the operated plots were surveyed using Real-Time Kinematic (RTK) positioning technology to obtain the actual field boundaries and measure the ground-truth operation areas. As shown in **Figure 18**, the area measurement of a harvested field parcel was carried out in Jiamusi, Heilongjiang Province, China, on October 31, 2024.

**Figure 19:**
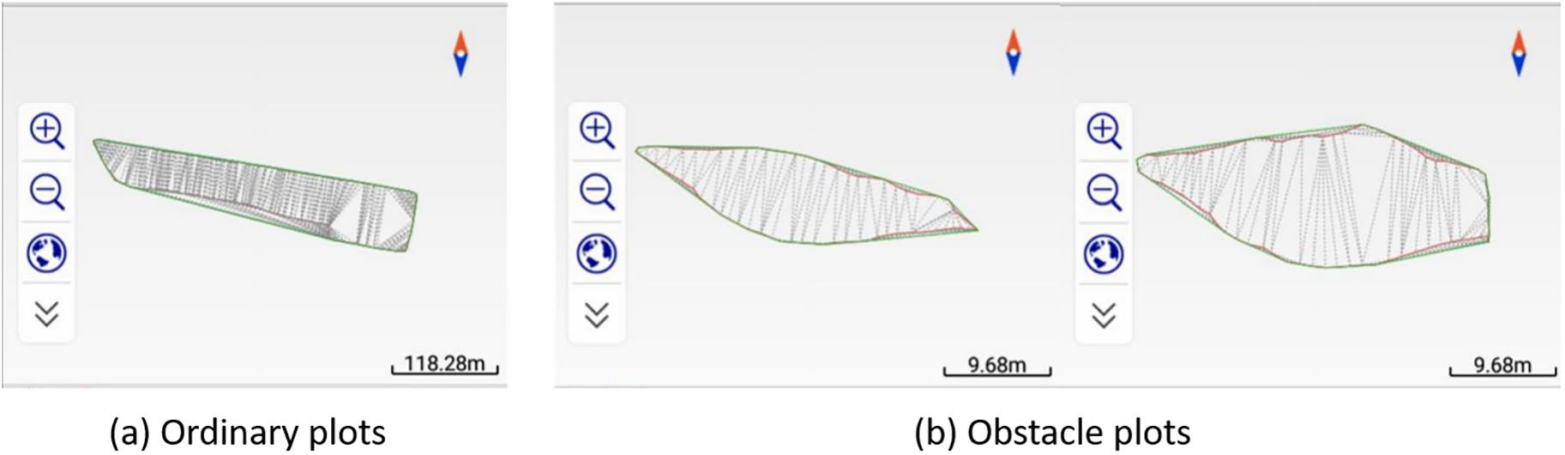
Operated plot processing. **(A)** Ordinary plots, **(B)** Obstacle plots.

For field validation, RTK was first used to survey the operated plot in order to obtain the actual field boundary and calculate the plot area. The non-operational regions within the plot were then identified and measured, and the corresponding blocked-area portions were excluded from the total area, as shown in **Figure 19**. On this basis, the agricultural machine carried out actual operations within the designated plot, while operational information such as terminal ID, implement width, operation start and end times, and operation type was recorded, thereby providing the basis for comparing the algorithm-estimated results with the field-measured results.

Field validation results are summarized in **Table 5**. The RTK-measured ground-truth area of the validation plot was 27.1165 mu. For wheat harvesting, the estimated area was 27.02 mu, corresponding to an area accuracy of 99.64% and a single-thread comprehensive processing efficiency of 5.30 mu/s. For rotary tillage, the estimated area was 27.14 mu, corresponding to an area accuracy of 99.91% and a processing efficiency of 5.12 mu/s. These results indicate that the proposed method can achieve high geometric consistency with RTK-measured reference areas in the tested field cases. However, because the RTK validation included only two operation cases in the same plot, the results should be interpreted as independent geometric verification rather than exhaustive validation across all machinery types and field conditions.

**Table 5:** Verification results.

Terminal	Width	Type	Start time	End time	Sreal	Sest	Acc	Time	Eff
8618810 52419729	2.75	Harvest	14:30:33	15:36:32	27.1165	27.02	99.64	5.1	5.30
8657710 56553274	2.4	Tillage	10:53:45	12:20:12	27.1165	27.14	99.91	5.3	5.12

### 3.4 Discussion

To further position the proposed method within the current state of research, **Table 6** compares representative studies on agricultural machinery trajectory identification and operation-area estimation. The comparison considers methodological characteristics, deployment capability, and application scope. Unlike existing studies that mainly focus on trajectory classification or area estimation independently, the proposed framework integrates trajectory identification, operation-area reconstruction, and cloud-platform deployment within a unified workflow.

**Table 6:** Comparison of representative studies related to agricultural machinery trajectory identification and operation-area estimation.

Study	Data type	Method	Area estimation	Deployment	Limitation
Lu et al., 2015	Machinery trajectory	Geometric/trajectory area method	Yes	No/limited	Sensitive to trajectory noise
Poteko et al., 2021	GNSS data	Operation-mode identification	No/partial	No	Does not reconstruct operation area
Zhai et al., 2024b	Machinery trajectory	Bagging-SVM	No/partial	Limited	Feature-dependent
Zhang et al., 2024	Machinery trajectory	Two-stage clustering	No/partial	Limited	Weak in complex mixed boundaries
Han et al., 2025	Machinery trajectory	Clustering + segmentation	Partial	Limited	Area reconstruction not emphasized
This study	Platform trajectory	Spatiotemporal grouping + CBGAM U-Net + spline buffer	Yes	Yes	RTK validation cases still limited

The results demonstrate that the staged framework can better balance recognition accuracy and deployment efficiency than using a single category of method alone. The reason is that different stages deal with different levels of uncertainty in the trajectory data. Preprocessing and spatiotemporal neighborhood grouping remove only clearly invalid or long-distance non-operational trajectory segments, thereby reducing the spatial span and noise level of trajectory images without making irreversible decisions for ambiguous mixed regions. The semantic segmentation model is then applied to candidate regions where local thresholds are insufficient, such as field-road intersections, road-transfer trajectories close to field boundaries, drifting trajectories, and sparse field-operation trajectories. This design reduces the computational burden of deep learning while preserving its advantage in extracting spatial morphology and contextual information.

The improvement obtained by CBGAM U-Net is mainly related to the representation characteristics of agricultural machinery trajectory images. Unlike natural images, trajectory images are sparse, line-shaped, and strongly dependent on local motion attributes. Operation and non-operation trajectories may be spatially adjacent or even partially overlapped in the pixel domain, especially near headlands and field-road intersections. By incorporating channel and spatial attention into the skip-connection stage, CBGAM U-Net enhances informative motion channels and critical spatial regions while retaining low-level boundary information from the encoder. This explains why the model improved the identification of road-transfer trajectories adjacent to field parcels with only a limited increase in inference time.

For operation area estimation, the main source of error is the propagation from trajectory-centerline uncertainty to polygon-boundary uncertainty. If invalid road-transfer trajectories are retained, buffer construction will produce significant overestimation. If valid sparse operation trajectories are removed, the recovered polygon will contain artificial missed areas. In addition, raw polyline buffering is sensitive to local angular changes, endpoint expansion, and positioning jitter. The cubic spline smoothing and compensation-correction strategy used in this study was intended to reduce these geometric artifacts. The outward width compensation improves coverage completeness when the recorded GNSS line deviates from the true implement centerline, while the subsequent inward boundary correction suppresses the systematic overestimation introduced by buffer expansion. Therefore, the area estimation accuracy depends not only on the buffer radius, but also on the reliability of valid trajectory extraction and the geometric stability of the reconstructed centerline.

Several limitations remain. First, although the IAEMP dataset covers multiple provinces and machinery types, the independent RTK validation in this study included only two operation cases in one validation plot. Additional RTK-measured parcels across different regions, crop types, field shapes, and machinery types are needed to further quantify the generalization ability of the method. Second, the current area estimation is performed in a planar projected coordinate system. For sloping farmland, the planar area may differ from the true surface area, and future work should incorporate digital elevation models or RTK-derived elevation information to support terrain-corrected area estimation. Third, weak GNSS signal conditions were treated mainly through preprocessing and abnormal-point filtering, but severe signal loss or long-duration drift was not fully solved in this study. Future research should integrate multi-source positioning quality indicators and uncertainty-aware trajectory reconstruction to improve robustness under poor signal conditions.

## 4 Conclusions

This study proposed a staged agricultural machinery operation trajectory identification and operation area estimation method for cloud-platform deployment. The method integrates trajectory preprocessing, spatiotemporal neighborhood grouping, multi-channel trajectory image construction, CBGAM U-Net semantic segmentation, cubic-spline-smoothed vector buffering, and inward boundary correction. The main conclusions are as follows:

1. The proposed multi-stage trajectory identification framework effectively combines point-level trajectory constraints, segment-level grouping, and pixel-level semantic segmentation. By removing evidently invalid trajectories before image construction and applying semantic refinement only to candidate mixed regions, the method improves the discrimination of field-operation trajectories, road-transfer trajectories, drifting trajectories, and field-road intersection trajectories while maintaining practical computational efficiency.
2. The CBGAM U-Net model improved the representation of sparse and line-shaped trajectory images by enhancing both channel-wise motion features and spatially critical regions in the skip-connection stage. Compared with the original U-Net, the proposed model improved trajectory identification performance with only a small increase in inference time, indicating its suitability for platform-level trajectory analysis.
3. The cubic-spline-smoothed vector-buffer area estimation method reconstructed operation coverage regions from refined trajectory centerlines. The combination of trajectory smoothing, width compensation, polygon union, and inward boundary correction reduced local boundary distortion and area bias caused by sparse sampling, repeated operation, pseudo-missed operation, and positioning deviation.
4. Platform-level validation using IAEMP historical operation records demonstrated the deployability of the proposed method for cloud-based agricultural machinery management. Independent RTK-based field validation further showed high area accuracy in the tested wheat harvesting and rotary tillage cases. Nevertheless, because the RTK validation cases were limited, future work should expand field validation across more regions, machinery types, field slopes, and weak-signal environments, and should incorporate terrain elevation information for slope-corrected area estimation.

## Data Availability

The original contributions presented in the study are included in the article/supplementary material. Further inquiries can be directed to the corresponding author.
